# Pericardial adipose tissue promotes transition to heart failure with reduced ejection fraction upon pressure-overload in mice

**DOI:** 10.1007/s00395-025-01116-x

**Published:** 2025-07-03

**Authors:** Yi Xuan Shia, Kathleen Pappritz, Anna Cristina Kaltenbach, Guo Li, Valentina Fardella, Sophie Van Linthout, Daniela Carnevale, Sabine Steffens, Sarah-Lena Puhl

**Affiliations:** 1 Institute for Cardiovascular Prevention (IPEK), Ludwig-Maximilians-Universität (LMU) Munich, Munich, Germany; 2 Comprehensive Heart Failure Center, University Clinic Würzburg, Würzburg, Germany; 3 BIH Center for Regenerative Therapies (BCRT), Berlin Institute of Health at Charité, Universitätsmedizin Berlin, Berlin, Germany; 4 German Center for Cardiovascular Research (DZHK), Partner Site Berlin, Berlin, Germany; 5 Research Unit of Neuro and Cardiovascular Pathophysiology, IRCCS Neuromed, Pozzilli, Italy; 6 Department of Medical and Surgical Sciences and Biotechnologies, “Sapienza” University of Rome, Rome, Italy; 7 German Center for Cardiovascular Research (DZHK), Partner Site Munich Heart Alliance, Munich, Germany

**Keywords:** Pressure-overload, HFrEF, Pericardial adipose tissue, Macrophages, Fibrosis

## Abstract

In patients, severity of pressure-induced heart failure (HF) due to aortic stenosis and metabolic disorder correlates with thickness and mass of epicardial adipose tissue (EAT). We examined the role of the less studied pericardial adipose tissue (PAT) during manifestation and progression of pressure-induced HF in mice. Progressive remodeling was assessed in C57BL/6 J males, aged 9 weeks, following sham surgery or transverse aortic constriction (TAC) for 1 week (early pressure-overload), 8 (chronic pressure-overload), or 12 weeks (HF with reduced ejection fraction, HFrEF) with or without concomitant PAT excision. PAT removal did not affect early (1-week TAC) or chronic (8 weeks) pressure-overload-induced concentric remodeling. However, initial PAT excision prevented lung congestion, progressive LV dilation and systolic dysfunction and thereby protected against transition to HFrEF. This protection was associated with alleviation of early TAC-induced pro-inflammatory monocyte and macrophage expansion, attenuation of persistent pro-hypertrophic, pro-inflammatory and pro-fibrotic LV gene expression and the reduction of microscar and perivascular fibrosis in the long term. The latter was reflected by reduced peri-coronary accumulation of pro-fibrotic CD206^+^ macrophages, and prevention of *periostin* upregulation. Moreover, PAT protein directly activated naïve cardiac fibroblasts in vitro while bulk RNAsequencing revealed the initiation of an extracellular matrix deposition, monocyte recruiting, and macrophage activation program in the PAT early upon TAC. Our data suggest that PAT does not exert crucial impact on pressure-induced hypertrophy, while its removal counteracts HFrEF manifestation in mice, at least in part, by preventing excessive fibrotic responses suggested to derive from reciprocal fibroblast-macrophage interactions.

**Supplementary Information:**

The online version contains supplementary material available at 10.1007/s00395-025-01116-x.

## Introduction

Heart failure (HF) can either manifest with preserved ejection fraction (HFpEF) or with reduced EF (HFrEF). HFpEF derives from sustained pressure-overload on the left ventricle (LV), hampering LV compliance and thereby leading to diastolic dysfunction. Predominant causes are hypertension, aortic stenosis, metabolic disorders, such as diabetes and obesity, and inherited hypertrophic cardiomyopathies. According to several clinical studies, establishment and severity of aortic stenosis-mediated heart failure and HFpEF can be predicted by enhanced epicardial adipose tissue (EAT) thickness and volume, deriving from adipocyte hyperplasia [11, 33, 34, 40, 53, 57, 61]. Contribution of EAT to HF progression involves predominantly its own metabolic remodeling resulting in reduced thermogenesis and fatty acid supply for the heart, and an endocrine switch toward release of pro-inflammatory cytokines such as Interleukin-6, and -1β (IL-6, -1β), tumor necrosis factor α (TNFα) and C-motif chemokine ligand 2 (CCL2), directly contributing to inflammation, fibrosis, microvascular dysfunction, and rarefaction of the stressed myocardium [9, 21, 23, 32, 40, 61, 64]. Therefore, targeted therapeutic alterations of the stress-responsive cardiac adipose tissue secretome could alleviate cardiovascular burden.

Cardiac fat encompasses different regional depots, namely pericoronary, paracardial, EAT, and pericardial adipose tissue (PAT), whereby the latter two represent the main depots [21, 69]. While EAT is located between the myocardium and the visceral pericardium, sharing microvasculature with the myocardium, the so far less investigated PAT is attached to the parietal pericardium [21, 24, 46]. While EAT is not found in rodents, PAT is also present in the murine thorax, positioned partly above the left coronary artery and supplied by the paracardiacophrenic artery and not by coronary arteries [1, 10, 22]. Yet, notably, mice have numerous pores in the pericardium which allow pericardial fluid to pass through and facilitate direct diffusion of adipose tissue-derived products into the myocardium [39].

Despite the emerging role of EAT and PAT as prognostic risk factors for cardiovascular diseases, in depth mechanistic examinations are rare, but also a potential role for EAT/PAT in HF not originating from metabolic disorders has barely been investigated so far [38]. Yet, in aortic stenosis patients, independent of diabetic burden, EAT thickness is reportedly even higher than in metabolic HFpEF scenarios and among different aortic stenosis groups the highest in high-flow high-gradient stenosis with reduced EF [11, 53]. An additional prognostic and potentially therapeutic evaluation of PAT during (the transition from HFpEF to) HFrEF in patients could crucially affect disease management of severe aortic stenosis. With respect to a further HFrEF scenario, Horckmans et al. have demonstrated that so-called fat-associated lymphoid clusters (FALCS) in the PAT are activated by acute myocardial infarction in mice and that surgical PAT removal exerts beneficial effects by mitigating excessive neutrophil infiltration and alleviating fibrosis of the infarcted heart [20]. We aimed at elaborating the impact of PAT on the pressure-overload-mediated remodeling and HF progression, rather mimicking aortic stenosis, in the absence of metabolic impairment and acute ischemia-driven inflammation. The inflammation of the pressure-overloaded heart is predominantly attributed to an early and transient immune response. Revelo et al. have demonstrated that leukocytes are expanding in the heart 1-week post-TAC with macrophages being by far the most prominently accumulating cell population [50]. Moreover, this distinct immune response had vanished again 4 weeks after TAC. Further studies have shown that LV expansion of reparative, and herein especially resident cardiac macrophages (RCM) is indispensable for adaptation of the heart to chronic stress, while recruited monocyte-derived (bone-marrow derived, BMDM), pro-inflammatory populations are driving maladaption [43, 45, 59, 70]. We hypothesized that PAT is also responsive to LV afterload and in turn modulates macrophage and fibrotic responses of the underlying myocardium and thereby determines LV remodeling, and functional outcome. To assess the impact of PAT on disease establishment and progression, we comprehensively examined the effect of a surgical PAT removal on early pressure-overload, chronic pressure-overload, and HFrEF induced via different time periods of transverse aortic constriction in mice.

## Methods

### Animal cohort

Male C57BL/6 J mice, aged 9 weeks, purchased from Janvier (Le Genest-Saint-Isle, France) were subjected to transverse aortic constriction (TAC) or sham surgery with (TAC-PAT; sham – PAT) or without (TAC, sham) an additional surgical PAT removal (n ≥ 10/group). A male cohort was chosen due to higher susceptibility of men to HFrEF [4]. All animal procedures were performed conform to the guidelines from Directive 2010/63/EU, the Government of Upper Bavaria (ROB-55.2.2532.Vet_02-19–59), the Landesamt für Gesundheit und Soziales Berlin (LaGeSo, T0025/15), the ARRIVE guidelines and the ethical standards laid down in the 1964 Declaration of Helsinki and its later amendments.

### TAC and PAT excision

A detailed description of the surgical procedures can be found in the article’s supplementary information. Briefly, mice were randomly assigned to either permanent banding of the aortic arch between the innominate artery and the right subclavian artery using a 7–0 thread and a 27 G place holder to induce LV afterload or to a sham operation, respectively. The severe constriction, determined by the 27 G place holder, was employed to model disease progression from LV hypertrophy with diastolic impairment to a phenotype with clearly reduced ejection fraction [51]. A subset of TAC and sham mice was assigned to an additional PAT excision. Herein, the third intercostal room was opened and the part of PAT, which is attached to the LV expanding from below the left atrium to the apex, was excised (Supplementary Information Fig. S1a). Surgeries were performed under deep anesthesia (medetomidine/midazolam/fentanyl; 0.5/5.0/0.05 mg/kg; intraperitoneally, i.p.). Anesthesia depth/surgical tolerance was confirmed by lack of blinking, whiskers movement, and toe withdrawal effect. Anesthesia was antagonized by flumazenil/atipamezol (0.1/0.5 mg/kg; i.p.). For analgesia, buprenorphine (0.05 mg/kg body weight; subcutaneously, s.c.) was injected immediately, 6 h, 24 h, and 32 h after surgery. In addition, meloxicam (0.2 mg/kg; s.c.) was administered 24 h after surgery.

### Echocardiography

All LV structural and functional imaging studies were performed using the Vevo® 3100 imaging system (VisualSonics; Scanhead: MX550D, 25–55 MHz, cardiac mouse) 1, 4, 8, and 12 weeks post-intervention. LV morphology and function was assessed as described previously under low-dose isoflurane anesthesia (1–1.5% isoflurane, 98.5–99% oxygen, flow rate 1 l/min) [48].

Extent of TAC-induced LV afterload was determined by aortic gradient measurements at the level of constriction in TAC mice within 48 h post-intervention (Scanhead: MX250S, 15–30 MHz, cardiac mouse).

### Tissue sampling and processing

At each study endpoint, mice were sacrificed by overdosed xylazine/ketamine (120/10 mg/kg body weight i.p.) anesthesia and subsequent exsanguination via right ventricular puncture and EDTA blood collection after confirmation of lack of reflexes, whiskers movement, and breathing. Organs/tissues for gravimetric, histological, and flow cytometry analyses were excised and processed as described previously [47]. Briefly, the myocardium was split for gene expression and histological analyses or for gene expression and flow cytometry analyses, respectively, depending on the post-TAC time-point and read-outs. LV transverse tissue slices for histology were fixed in 4% paraformaldehyde or optimal cutting temperature (OCT) and stored at 4 °C or -20 °C, respectively. PAT of sham and TAC-subjected mice was excised for subsequent flow cytometry, histological analyses, or RNAsequencing. Tissue for qPCR was immediately frozen in liquid N_2_ and stored at -80 °C. Tissue for flow cytometric analyses was directly transferred into PBS and stored on ice until further processing.

### Flow cytometry

Heart tissue, EDTA blood, bone marrow cells, and spleens were processed as described previously [47]. PAT samples were subjected to the same processing protocol as cardiac tissue. Data were acquired on a FACS Canto II and Fortessa (BD Biosciences), and analyzed using FlowJo software (Ashland, USA). List of antibodies used, and the applied gating strategies can be found in the supplementary information.

### Histology

In paraffin-embedded transverse midventricular tissue slices (5 µm), cardiomyocyte diameter was assessed following hematoxylin and eosin (H&E) staining while fibrosis was assessed following Sirius red staining, as described previously [47]. Total LV collagen content was quantified as Sirius red stained tissue percentage per LV transverse section. The papillary muscles exhibited microscar foci which were calculated as percentage collagen of the respective endocardial area [12, 14, 56]. For perivascular fibrosis assessment, individual images (10 × magnification) containing the left coronaries were taken and for perivascular fibrosis quantification, the percentage of collagen around three arteries per LV section was averaged and normalized against the vessel area. Analyses were performed using ImageJ software.

Detailed protocols for immunohistochemistry can be found in the article’s online supplementary information. In brief, for tyrosine hydroxylase (TH) staining, PAT was fixed, permeabilized, blocked, and stained with anti-TH and anti-perilipin-1. Tile scans were acquired in 10 × magnification. The total length of sympathetic TH + nerves was quantified using LAS X software.

For α-SMA and macrophage staining, acetone-fixed and -permeabilized LV transverse OCT sections were blocked and stained with anti-α-SMA, or anti-α-actinin, anti-CD68 and CD206, respectively. Nuclei were stained with Hoechst. Myofibroblasts were manually counted (Image J) from tile scans of the whole section acquired in 10 × magnification, herein a α-SMA + signal that surrounded a nucleus was identified as an individual cell, i.e., a myofibroblast, and α-SMA + signals that clearly indicated the border of a lumen, suggestive of vessels and thereby vascular smooth muscle cells, were excluded from the analyses. Macrophage populations were manually counted (Image J) in 8 fields of view (FoV) per section, acquired in 20 × magnification, and averaged per section. CD68 + CD206 + and CD68 + CD206- sub-populations were quantified as percentage of CD68 + cells.

### LV mRNA expression analyses

For qPCR analysis of whole cell lysates, tissue was homogenized, lysed, and RNA isolated using the Qiagen RNeasy Kit. PCRs were run in duplicates with 10 ng/µl cDNA. mRNA expression was normalized per sample against Gapdh and relative gene expression was calculated using the ∆∆CT method. Data are expressed as fold over sham. TaqMan probes, purchased from Thermo Scientific, are listed in the supplementary information.

### ELISA

Plasma was collected from EDTA blood after centrifugation at 10,000 rpm for 10 min, snap-frozen in liquid N_2_ and stored at -80 °C. For norepinephrine (NE) quantification, thawed plasma was treated with catecholamine stabilizing solution (1 mM EDTA, 4 mM sodium metabisulfite, pH 7.5 diluted 1:4 in distilled water) and detected via an ultra-sensitive ELISA (BA E-5200, Labor Diagnostika Nord, LDN, Germany) according to the manufacturer's instructions at 450 nm (Infinite 200 Pro, Tecan). TGF-β1 was quantified in thawed samples (1:3 dilution) via the DuoSet ELISA (DY1679-05, R&D system, Minneapolis, MN, USA) at 450 nm (Infinite 200 Pro, Tecan).

### RNA sequencing

PAT was lysed with 27.8 µl Quiagen RLT Plus lysis buffer (#1,053,393) + 1% β-mercaptoethanol per mg PAT via bead homogenization to yield an RNA concentration of 2–20 ng/µl following the RNA extraction with Zymo Direct-zol RNA MicroPrep (#R2062). Extracted RNA was sent for bulk RNA-seq [25]. Differential gene expression was calculated using DESeq2 Bioconductor package in R version 4.1.2 under Ubuntu 20.04.3 LTS system, based on the negative binomial distribution [30]. Genes were filtered using a custom function to select genes expressed in at least 10% of samples [25]. Size factors and dispersion of samples were estimated first, then Negative Binomial GLM fitting, and Wald statistics calculation was performed by DESeq2. Differentially expressed genes (DEG) were identified by the following criteria: adjusted P value < 0.10 and fold change > 2, based on the calculation results of DESeq. Gene ontology enrichment analysis and KEGG analysis were performed using the clusterProfiler Bioconductor package2 with DEGs (only filtered by adjusted P value < 0.10). Cneplots and dotplots were drawn by enrichplot package [62, 68].

### Cell culture

To elaborate whether PAT from failing hearts exhibit pro-fibrotic signatures, cardiac fibroblasts (cFBs) were obtained from 12-week-old C57BL/6 J males (Charles River, Sulzfeld, Germany), as described previously [42], exposed to PAT protein extracted from naïve mice or after 12-week TAC, respectively, and characterized based on cell viability (formazan reduction), proliferation (crystal violet staining), and collagen production (Sirius red staining) [41]. The detailed protocol is provided in the online supplementary methods.

### Study design and statistical analyses

In vivo part of the study: Experimental animals were randomly assigned to treatment groups. Group sizes were estimated based on power analysis of LV EF as functional read-out and LV *Ctgf* expression as a molecular fibrosis marker with an alpha error of 5% and a power of 80%. To detect a 20% change in EF with an expected standard deviation (SD) of 10%, an experimental group size of n = 3 was estimated. To detect a 100% increase in *Ctgf* with a 45% SD n = 4 mice were required to fully power the study. All experimental data analyses were performed in a blinded manner. mRNA expression data are presented relative to control (i.e., sham mean ± SE). Replicates were treated as n = 1 sample and the declared group size is the number of independent values subjected to statistical analysis. Gravimetric heart data were collected from all experimental animals employed in this study including the sham and TAC cohorts serving for 1 and 12 weeks PAT RNAsequencing, resulting in varying group sizes herein (1 week: *n* = 9–17/group; 8 weeks: *n* = 11–15/group; 12 weeks: *n* = 12–18/group), while echocardiography was acquired only in a subset of comparable group sizes (1 week: *n* = 8–11/group; 8 weeks: *n* = 11–12/group; 12 weeks: *n* = 10–13/group). Flow cytometry, histology, and mRNA analyses were performed in subgroups of the overall study population due to tissue limitations. Exact group sizes per experimental approach and time-point investigated are listed in the online supporting material (Supplementary Information Table S3). Continuous data are presented as mean ± SE. Individual values are plotted for all acquired data except for echocardiographic and gravimetric time courses to facilitate visual perception of the respective data (i. e. 12–16 groups with *n* ≥ 8/group). For multiple group comparisons involving two independent variables, two-way ANOVA was applied with Sidak’s (four groups) or Tukey’s post hoc test (8–16 groups), as appropriate. To determine differences between two groups, unpaired student’s *t* test was performed after passing normality testing. All calculated p values are two-sided. Differences with a *p* < 0.05 were considered statistically significant. All analyses were performed with Microsoft Excel 2010 and GraphPad Prism 8.0 and 9.0 software. Exact p values for each statistical comparison performed can be found in the online supplementary information (Supplementary Information Table S3). In vitro part of the study (cFB): Data acquired from in vitro experiments with cFB were first tested for normal distribution using the Shapiro–Wilk test. For non-parametric data, Kruskal–Wallis test with uncorrected Dunn’s post hoc test was performed. For parametric data, Brown–Forsythe and Welch-ANOVA followed by unpaired t test with Welch’s correction was applied.

## Results

### Impact of PAT removal on pressure-overload-induced LV hypertrophy

**Fig. 1:**
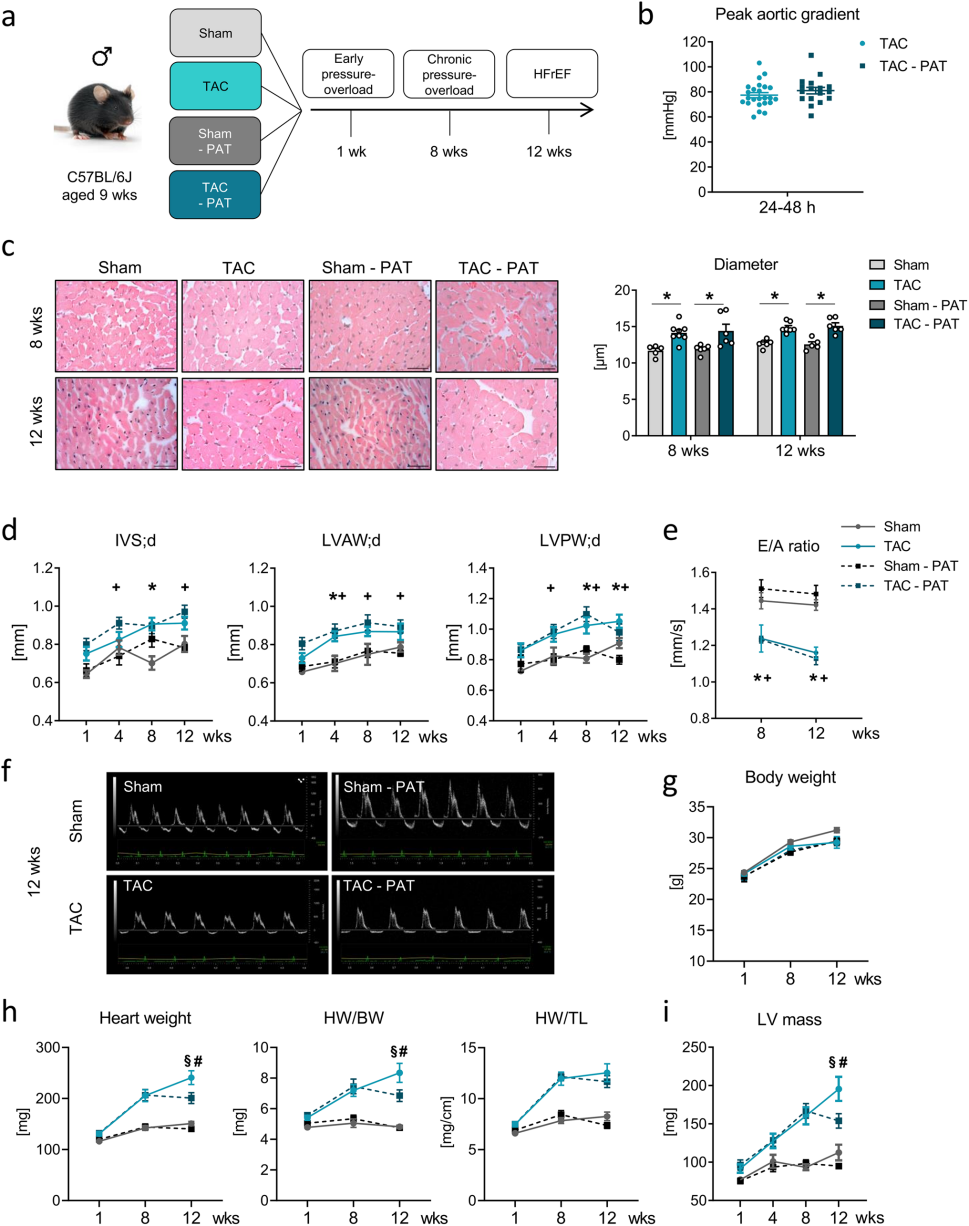
Impact of PAT removal on pressure-overload-induced LV remodeling. **a** Mice were randomly assigned to sham or TAC surgery without (sham, TAC) or with additional PAT removal (sham-PAT, TAC-PAT) for 1, 8, or 12 weeks, reflecting early establishment of pressure-overload, chronic pressure-overload, and a HFrEF stage. **b** Peak aortic gradient assessed via echocardiography in TAC and TAC – PAT mice 24–48-h post-intervention (*n* = 17–24). **c** Representative hematoxylin and eosin-stained midventricular cross-sections. 8 and 12 weeks post-intervention (40 × magnification, scale bar 15 µm) and quantification of cardiomyocyte diameter (*n* = 5–8/group; *p < 0.05). **d** End-diastolic interventricular septum (IVS;d), LV anterior (LVAW;d), and posterior wall (LVPW;d) thickness 1, 4, 8, and 12 weeks post-intervention determined via echocardiography (*n* = 8–13/group). **e** E/A ratio assessed via echocardiography 8 and 12 weeks post-intervention (*n* = 7–9/group). **f** Representative mitral valve flow Doppler recordings 12 weeks post-TAC and -sham, respectively. **g** BW and **h** HW per se and normalized against BW and TL, 1, 8, and 12 weeks post-intervention (*n* = 9–18/group; evident significant differences between TAC cohorts and their corresponding sham groups at each time-point measured and between 8 and 12 weeks TAC cohorts and their corresponding 1-week time point not indicated). **i** Calculated LV mass 1, 4, 8, and 12 weeks post-intervention (*n* = 8–13/group; evident significant differences between TAC cohorts and their corresponding sham groups at each time-point measured and between 8- and 12-week TAC cohorts and their corresponding 1-week and 4-week time point not indicated). Bars/symbols indicate mean ± SE; two-way ANOVA with Sidak’s post hoc test or Tukey’s post hoc test, as appropriate; *p* < 0.05; * vs. sham; + vs. sham-PAT; # vs. TAC-PAT; § vs. corresponding 8-week cohort

To assess the impact of PAT on pressure-overload-induced remodeling, male WT mice were subjected to sham or TAC surgery without (sham, TAC) or with additional PAT removal (sham-PAT, TAC-PAT) for 1, 8, or 12 weeks, reflecting early establishment of pressure-overload, chronic pressure-overload, and a HFrEF stage (Fig. 1a). Initial peak aortic gradient measurements confirmed a comparable extent of LV afterload in the two TAC groups before subsequent characterization of the study cohorts (Fig. 1b). Regarding LV hypertrophy, PAT removal did neither affect TAC-triggered concentric cardiomyocyte growth (Fig. 1c) nor diastolic LV wall thickening (Fig. 1d), resulting in similarly impaired mitral valve flow, as approximate measure for impaired LV compliance, in both TAC groups during long-term pressure-overload (Fig. 1e, f). As expected, TAC led to a clear progressive increase in heart weight per se and normalized against body weight over time until the 12-week study endpoint and compared to sham conditions without any impact on time-dependent body weight gain (Fig. 1g, h and supplementary information Tables S1, S2). Initial PAT removal, however, halted and stabilized TAC-triggered hypertrophic heart growth at 8 weeks post-TAC (Fig. 1h and Supplementary Information Tables S1, S2) resulting in significantly lower heart weight (HW) and HW/ body weight (BW) ratios compared to the TAC group at the 12-week time-point. Even though PAT impact on HW to tibia length (TL) ratio was less pronounced, LV mass calculated based on echocardiography emphasized further a maladaptive hypertrophy limiting effect byPAT removal (Fig. 1i). Of note, neither TAC nor time-period post-surgery affected PAT weight (Supplementary Information Fig. S1b).

### Impact of PAT removal on pressure-overload-induced LV dilation and dysfunction

**Fig. 2:**
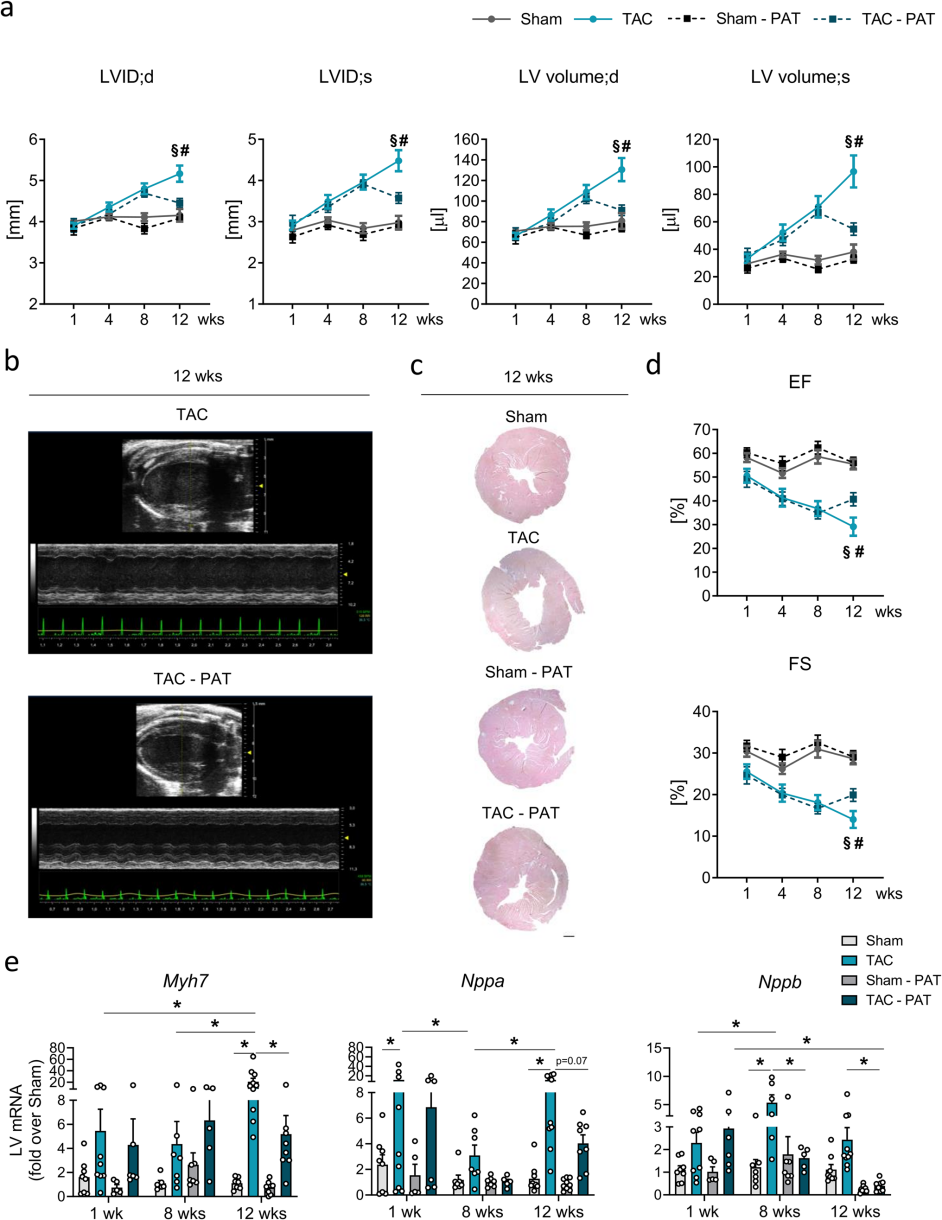
Impact of PAT removal on pressure-overload-induced dilation and systolic dysfunction. **a** End-diastolic and end-systolic LV internal diameter and volume 1, 4, 8 and 12 weeks post-intervention determined via echocardiography (n = 8–12/group; p < 0.05; # vs. TAC – PAT; § vs. 8 weeks; evident significant differences between TAC cohorts and their corresponding sham groups at each time-point measured and between 8- and 12-week TAC cohorts and their corresponding 1-week time point not indicated). **b** Representative B- and M-mode recordings of TAC and TAC–PAT LVs 12 weeks post-TAC. **c** Representative hematoxylin and eosin-stained midventricular cross-sections-. 12 weeks post-intervention (25 × magnification, scale bar 500 µm). **d** EF and FS 1, 4, 8, and 12 weeks post-intervention determined via echocardiography (*n* = 8–13/group; *p* < 0.05; # vs. TAC-PAT; § vs. corresponding 8-week cohort; evident significant differences between TAC cohorts and their corresponding sham groups at each time-point measured and between 8- and 12-week TAC cohorts and their corresponding 1-week time point not indicated). **e** LV mRNA expression of fetal genes 1, 8, and 12 weeks post-intervention relative to sham data of the respective time-point (*n* = 6–9; *p < 0.05). Bars/symbols indicate mean ± SE; two-way ANOVA with Tukey’s post hoc test

In agreement with the gravimetric observations, PAT removal also prevented pressure-overload-mediated progressive LV dilation beyond the 8-week phenotype while TAC per se triggered a time-dependent enlargement of diastolic and systolic diameter and volume with the former reflecting dilation and the latter hinting toward an impaired systolic contraction (Fig. 2a). The PAT impact on LV cavity size also becomes evident in the representative B- and M-mode recordings and LV transverse sections acquired 12 weeks post-TAC (Fig. 2b-c). The protection against progressive dilation resulted in the prevention of further systolic functional decline from 8 to 12 weeks post-TAC in the absence of PAT (EF and fractional shortening (FS) Fig. 2d), i.e., PAT removal prevented the transition into a clear HFrEF phenotype upon chronic pressure-overload. An aggravated HF stage in mice with intact PAT could be further confirmed by their clear signs of lung congestion which were absent in the TAC–PAT group at the 12-week follow-up examination (Supplementary Information Table S1 and Fig. S1c).

Moreover, in the presence of PAT, early (1-week TAC) indicated re-expression of fetal genes was promoted by TAC duration (*Myh7, Nppa*, *Nppb*), while the absence of PAT significantly attenuated the pro-hypertrophic response in the long term (12 weeks) or even led to a regression of the initial cellular response over time (*Nppb*; Fig. 2e). These data confirm prolonged prominent re-expression of fetal genes to be associated with HF progression and further emphasize that surgical PAT removal alleviates hallmarks of pathological hypertrophy such as stable high expression of the LV filling pressure marker *Nppb*. Of note, we could not find an evident PAT-dependent regulation of pro-hypertrophic phosphatase calcineurin or kinases, such as mitogen-activated protein kinases, AKT or Ca^2+^/calmodulin-dependent protein kinase II (data not shown).

### Impact of PAT removal on pressure-overload-associated LV inflammation

**Fig. 3:**
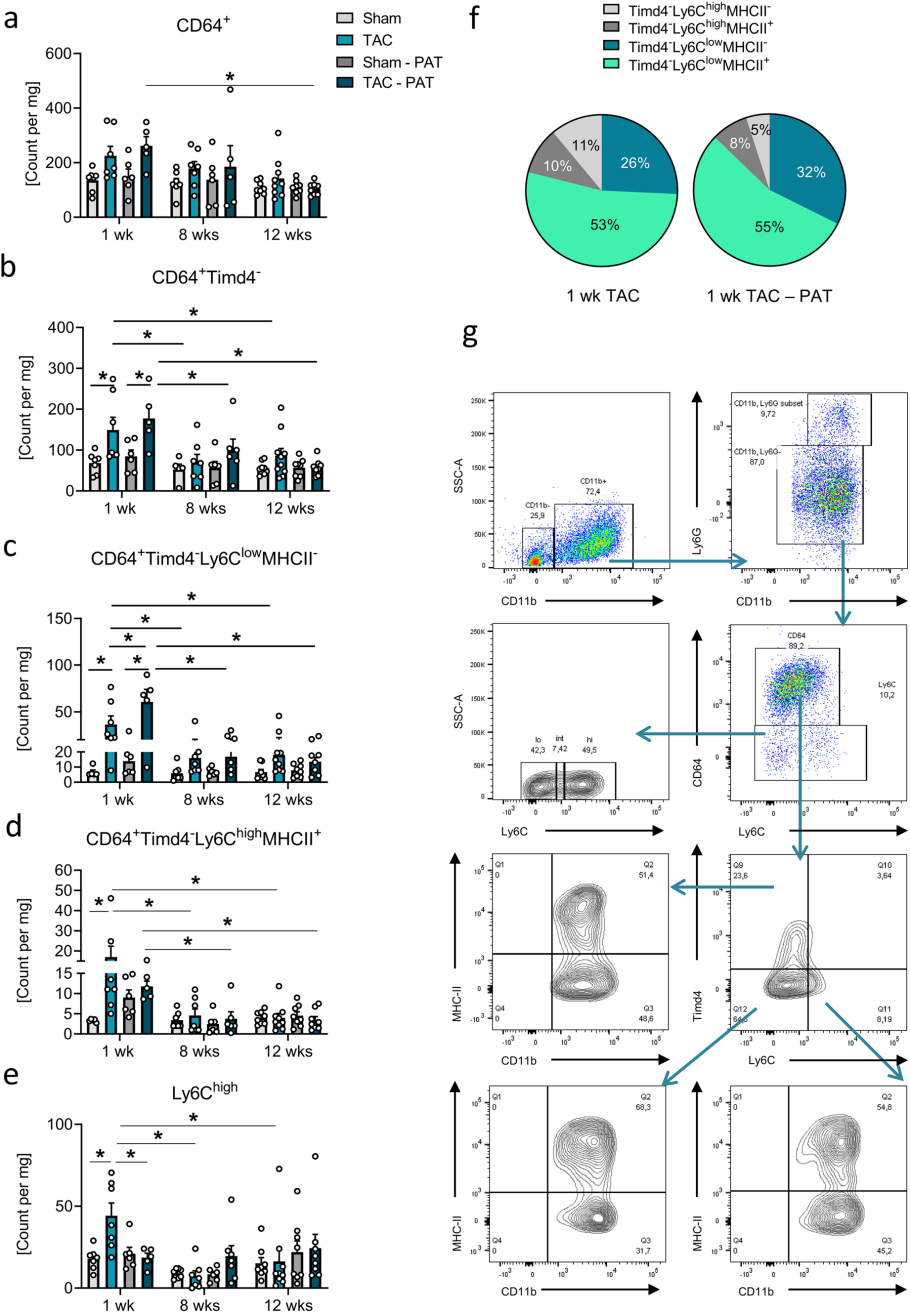
Impact of PAT removal on pressure-overload-associated LV inflammation. **a** CD64 + , **b** Timd4^−^, and herein **c** reparative Timd4^−^Ly6C^low^MHCII^−^, and **d** recruited pro-inflammatory Timd4^−^Ly6C^high^MHCII + macrophages, and **e** pro-inflammatory Ly6C^high^ monocytes per mg heart 1 week (*n* = 5–7/group), 8 weeks (*n* = 6–7/group), and 12 weeks (*n* = 7/group) post-intervention. Bars indicate mean ± SE; two-way ANOVA with Tukey’s post hoc test; *p < 0.05. **f** Percentage distribution of reparative and inflammatory BMDMs 1-week TAC vs. TAC-PAT. **g** Flow cytometry gating strategy to identify the respective sub-populations

The pressure-overloaded heart is further characterized by a subtle, transient LV inflammation, which can determine the extent of LV remodeling and consequently functional outcome. Revelo et al. have demonstrated macrophages to be the most prominently upregulated immune cell population in male BL/6 J hearts 1-week post-TAC, and that this response fully vanishes again 4 weeks post-TAC [50]. Kinetics of LV immune cell counts confirmed myeloid (CD11b^+^) and herein macrophage but not neutrophil expansion mainly after 1-week TAC (Fig. 3 and Supplementary Information Fig. S2a, b). While this was not associated with an evident accumulation of Timd4^+^ RCMs (Supplementary Information Fig. S2c, d), early pressure overload promoted a significant expansion of Timd4^−^ macrophages independent of PAT (Fig. 3b), which receded under chronic cardiac stress (8 and 12 weeks post-TAC). Interestingly, PAT removal potentiated the early accumulation of pro-reparative Timd4^−^Ly6C^low^MHCII^−^ subsets (Fig. 3c), while the presence of PAT promoted the transient recruitment of pro-inflammatory BMDMs (Timd4^−^Ly6C^high^MHCII^+^; Fig. 3d) and their precursor monocytes (Ly6C^high^; Fig. 3e) as well as *Il-1β* expression (Supplementary Information Fig. S2g). Consequently, removal of PAT was associated with a general macrophage shift in favor of reparative subsets (13% Ly6C^high^ macrophages in TAC-PAT vs. 21% in TAC, and 87% Ly6C^low^ macrophages in TAC-PAT vs. 79% in TAC, respectively; Fig. 3f and Supplementary Information Fig. S2e, f, with subsets identified according to Fig. 3g). These observations hint for the first time toward a role for PAT in regulating cardiac macrophage responses to pressure-overload in vivo. This PAT effect, however, seemed independent of the LV expression of monocyte attracting CC-chemokine ligand 2 (*Ccl2*), its receptor C–C motif chemokine receptor 2 (*Ccr2*), or of granulocyte–macrophage colony-stimulating factor (*Gm-csf*, Supplementary Information Fig. S2g). Of note, while the presence of PAT modulated the inflammatory response of the pressure-overloaded heart, the LV afterload in turn did not alter myeloid cell composition of PAT during disease progression (Supplementary Information Fig. S3).

Flow cytometry analyses after 1- and 8-week TAC were not capturing an evident increase of circulating myeloids, yet the 12-week time point was surprisingly associated with increased neutrophil levels over sham and the respective 8-week level in the TAC but not in the TAC–PAT group (Supplementary Information Fig. S4a–d). Whether this observation indicates a hallmark of HFrEF remains speculative. Of note, independent of pressure-overload, PAT removal was associated with increased monocyte counts in the bone marrow 1 week post-surgery and a similar trend could be concomitantly observed in the spleen, an effect which vanished again at 8 weeks (Supplementary Information Figs. S4e-h and S5a-d). Whether this reflects promoted (emergency and extramedullary) monopoiesis as a compensatory mechanism for the loss of the PAT as an immune cell depot itself or whether PAT removal limits the egress from hematopoietic stores and subsequent recruitment of monocytes to the side of injury, therefore explaining the limited LV BMDM infiltration, remains to be elucidated.

### Impact of PAT removal on pressure-overload-associated sympathetic impairment

**Fig. 4:**
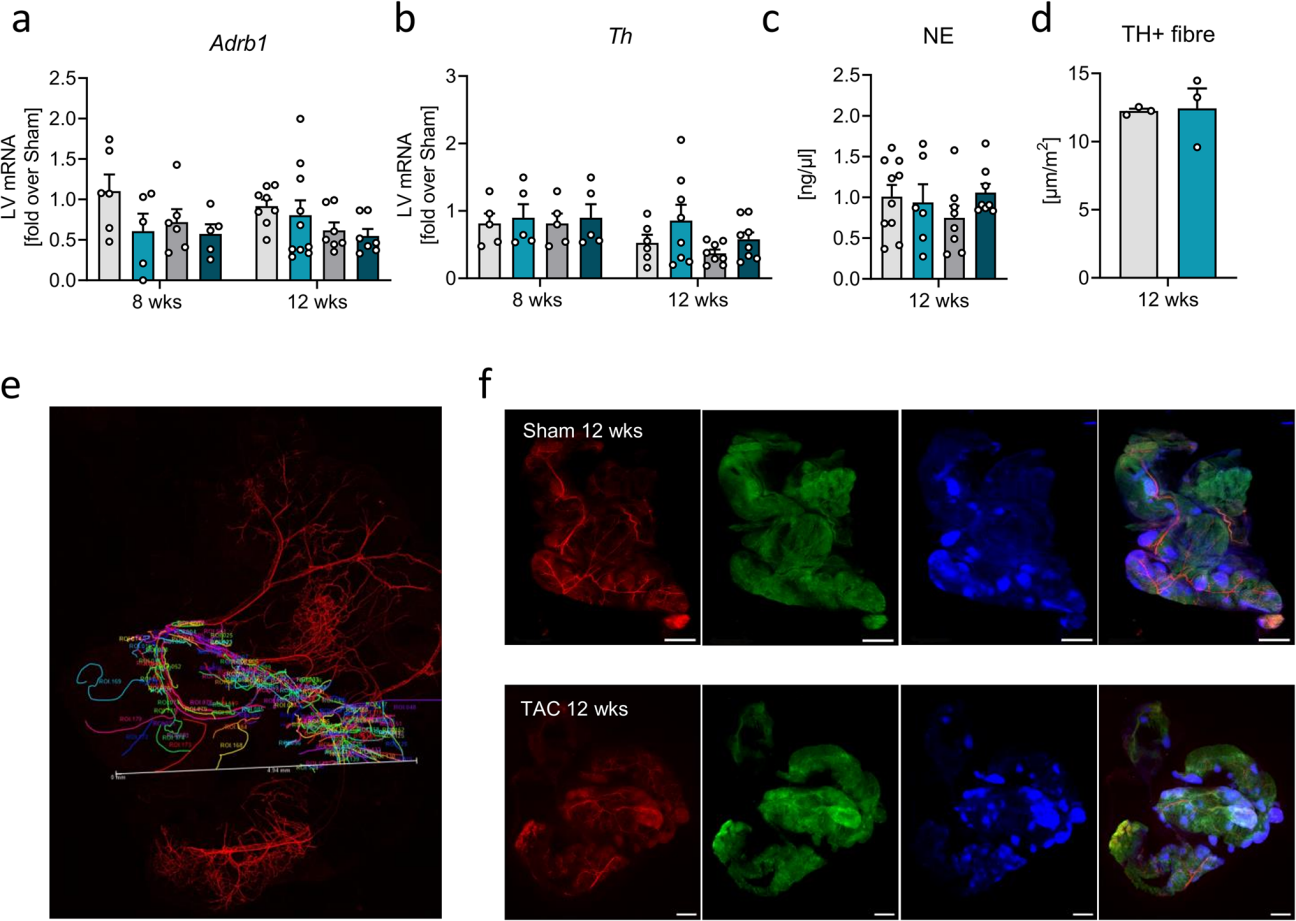
Impact of PAT removal on pressure-overload-associated sympathetic responses. **a** *Adrb1* and **b** tyrosine hydroxylase (*Th*) expression 8 and 12 weeks post-intervention fold over sham data per time-point investigated (*n* = 5–8/group). Two-way ANOVA with Sidak’s post hoc test. **c** Norepinephrine plasma level assessed via ELISA 12 weeks post-intervention (n = 5/group). **d** TH + fiber length in PAT 12 weeks post-TAC and sham, respectively, normalized against PAT area (*n* = 3/group; unpaired student’s *t* test). **e** Representative image of PAT stained against TH displaying the manual fiber length measurement performed. **f** Representative images from PAT excised 12 weeks post-surgery and stained against TH (red) and perilipin (green; 10 × magnification, scale bar: 1 mm), Nuclei were stained with Hoechst. Bars/symbols indicate mean ± SE

We have observed previously that LV adaption to pressure-overload is crucially determined by the type of macrophages expanding in the heart and that this beneficial macrophage response is tightly regulated by autonomous nervous system signaling through the spleen [45]. Moreover, chronic afterload and the subsequent LV remodeling are characterized and aggravated by reduced β-adrenoceptor sensitivity. Therefore, we assessed whether PAT removal could impact on LV function by influencing sympathetic factors. LV mRNA level of *Adrb1* and tyrosine hydroxylase (*Th*), and plasma noradrenaline (NE) level were not evidently affected by PAT presence or long-term pressure overload (Fig. 4a-d). We also examined whether cardiac afterload in turn affects PAT innervation extent but could not observe any differences in TH positive fiber lengths between PAT from 12-week TAC vs. 12-week sham mice (Fig. 4e-f). These analyses suggest that the PAT-mediated effects on LV maladaption are achieved rather independent of autonomous nervous system activation.

### Impact of PAT removal on pressure-overload-triggered fibrosis

**Fig. 5:**
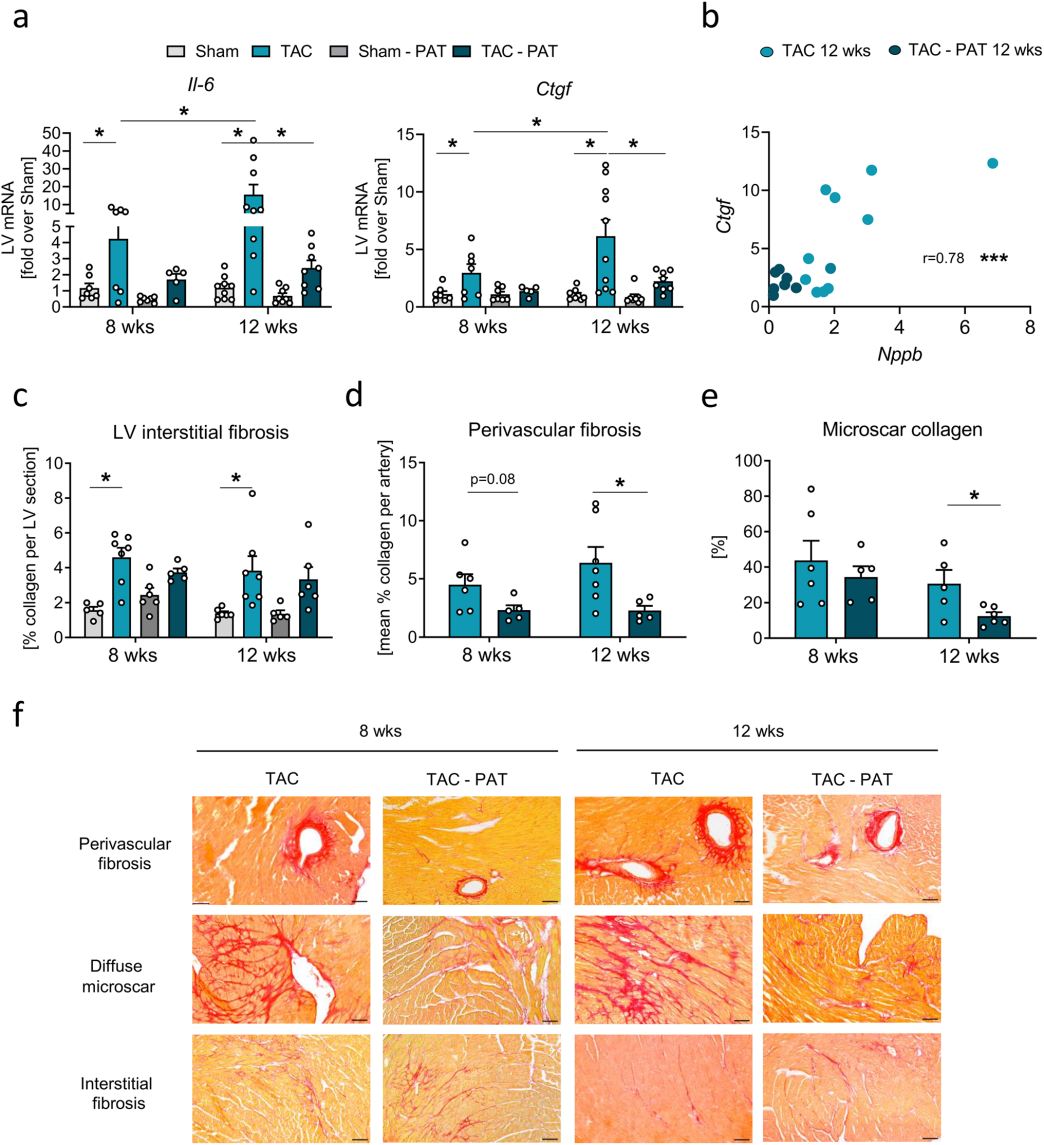
Impact of PAT removal on pressure-overload-triggered fibrosis. **a** LV mRNA expression of pro-fibrotic *Il-6* and *Ctgf* 8 and 12 weeks post-intervention relative to sham data of the respective time point (*n* = 5–9/group). **b** Correlation calculated via regression analyses between *Ctgf* and *Nppb* LV mRNA expression 12 weeks post-TAC (*n* = 5–9/group). **c** LV interstitial fibrosis 8 and 12 weeks post-intervention assessed via quantification of Sirius red stained fibers (*n* = 5–7/group). Quantification of **d** perivascular fibrosis and **e** diffuse microscars at the papillary muscle level 8 and 12 weeks post-TAC (*n* = 5–7/group). Bars/symbols indicate mean ± SE; two-way ANOVA with Sidak’s post hoc test; *p < 0.05. **f** Representative images of Sirius red-stained midventricular cross-sections. 8 and 12 weeks post-TAC, acquired at coronary level in the LVAW (top row), papillary muscles (middle row) and LVPW (bottom row; 10 × magnification, scale bar 100 µm)

The afterload-induced pathological remodeling process mainly encompasses maladaptive hypertrophy, recruitment and expansion of immune cells, and activation of, and collagen production by, fibroblasts, culminating in the functional deterioration of the heart. We therefore, looked next into the impact of PAT on the fibrotic response of the mechanically stressed heart. Interestingly, initial PAT removal almost completely blunted the progressive TAC-triggered LV upregulation of the pro-inflammatory and pro-fibrotic interleukin-6 (*Il-6*) and of connective tissue growth factor (*Ctgf*) (Fig. 5a). Moreover, *Ctgf* expression clearly correlated with transcription level of the heart failure marker *Nppb* 12 weeks post-TAC, resulting in an evident clustering of the TAC vs. TAC-PAT groups (Fig. 5b). This, in turn, underpins the beneficial impact of PAT removal on the progression of heart failure. Simultaneously, only in the presence of intact PAT, TAC clearly increased *Col3α1* expression over sham level which was reflected by a similar *Col1α2* expression pattern and reduced *Mmp-9* expression compared to the earlier disease stage (Supplementary Information Fig. S6a-c), suggesting potentiated ECM deposition and decreased ECM degradation at the HFrEF stage. Of note, lack of PAT impact on extent of interstitial fibrosis, LV *Mmp-2, Tgf-β*, and *Sox9* expression might distinguish the pro-fibrotic role of the fat depot in mechanically induced cardiac stress from its impact on fibrosis accompanying metabolic disorders which include free fatty acid infiltration into the heart (Fig. 5c and Supplementary Information Fig. S6d-f) [46].

Yet, further discrimination between types and location of fibrosis revealed that the absence of PAT limited microscar formation at the papillary muscle level and protected against perivascular fibrosis during progressive remodeling (Fig. 5d-f). These data could hint toward maintained cardiomyocyte coupling and improved myocardial perfusion/diffusion in the absence of PAT which physiologically overlays the left coronaries. Vice versa perivascular fibrosis associated with the presence of PAT is likely to exert perivascular compression, leading to functional impairment and reduced vascular flow contributing to LV functional deterioration [18].

### Impact of PAT removal on pro-fibrotic cell accumulation upon chronic pressure-overload

**Fig. 6:**
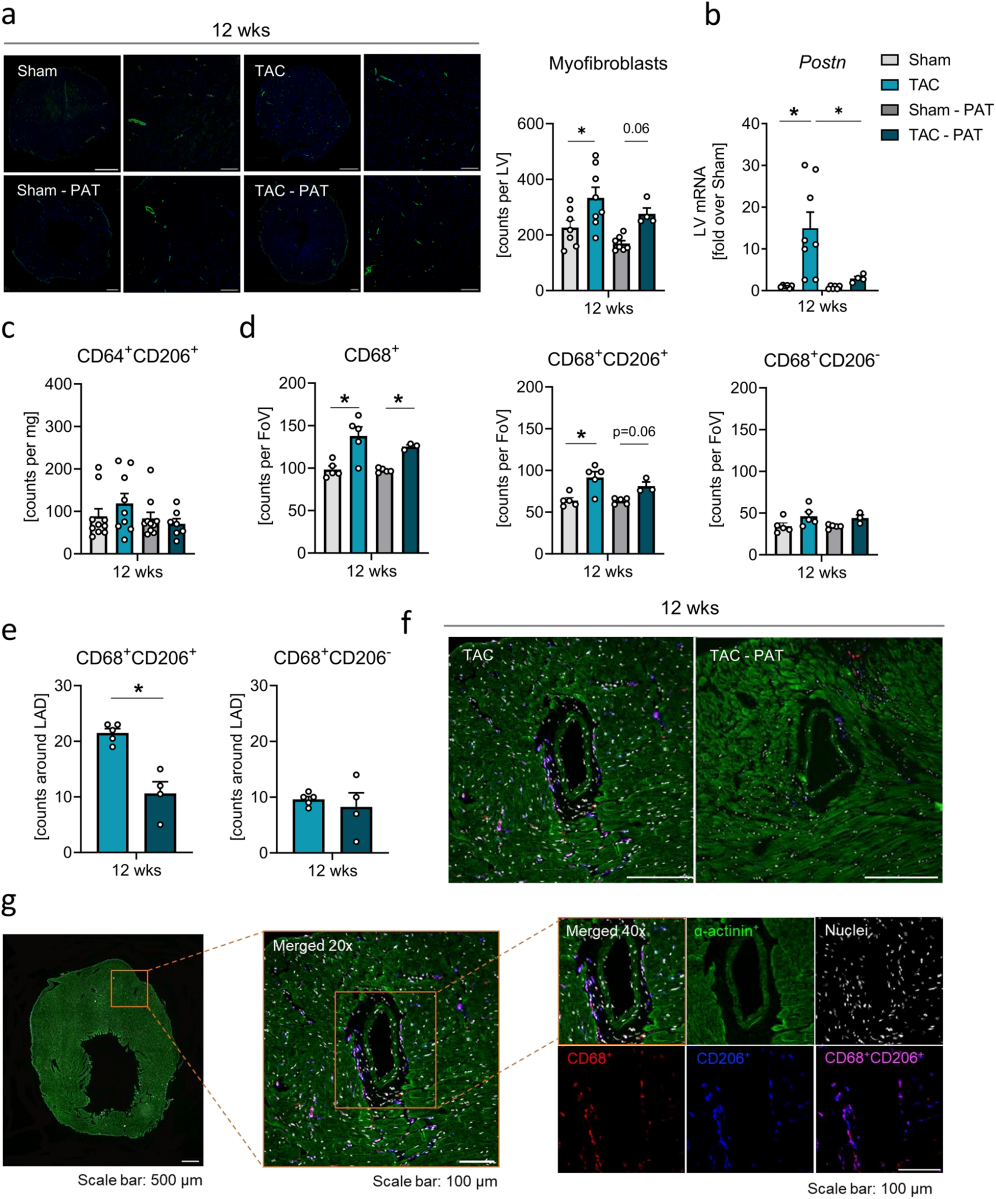
Impact of PAT removal on pro-fibrotic cell accumulation upon chronic pressure-overload. **a** Representative images of α-smooth muscle actin (α-SMA) stained (green; FITC conjugated mouse anti-α-SMA) transverse midventricular cryo-sections 12 weeks post-intervention. Nuclei are stained with Hoechst (tile scans (left panels), scale bar 1 mm; 10 × magnification (right panels): scale bar 200 µm) and quantification of positive cells per section (n = 4–8/group). **b** LV *Postn* mRNA expression 12 weeks post-intervention relative to sham (n = 4–8/group). **c** Cardiac CD64^+^CD206^+^ macrophage counts per mg tissue 12 weeks post-TAC (*n* = 7–11/group). **d** Averaged counts of overall CD68^+^ macrophages and herein of CD68^+^CD206^+^ and CD68^+^CD206^−^ macrophages per field of view (8 FoV per section, 20 × magnification, scale bar 500 µm) 12 weeks post-intervention (*n* = 3–5/group); two-way ANOVA with Sidak’s post hoc test; *p < 0.05. **e** Pericoronary quantification of CD68 + CD206 + and CD68 + CD206- macrophages 12 weeks post-intervention (n = 4–5/group); student’s *t* test. Bars/symbols indicate mean ± SE. **f** Representative images of transverse peri-coronary regions stained against α-sarcomeric actinin (green), CD68 (red) and CD206 (blue), double-positive CD68^+^CD206^+^ appear lilac. Nuclei (white) are stained with Hoechst (20 × magnification, scale bar 50 µm). **g** LV section with merged channels acquisition, and indication of the coronary area by orange framing (left panel, 5x) and zoomed-in merged image of the coronary area, indicated by orange framing (middle panel, 20x), further zoomed-in merged image, indicated by orange framing (right panel, 40x), and acquisitions of separate channels (green; mouse anti-α-actinin, secondary antibody: FITC donkey anti-mouse; red: rat anti-mouse CD68, secondary antibody: Alexa Fluor 647 Donkey anti-rat IgG, and blue: goat anti-mouse CD206, secondary antibody: Rhodamine Red-X Donkey anti-goat, white: Hoechst stained nuclei)

We next sought for the cell type sensitive to potential pro-fibrotic signals deriving from the PAT and thereby responsible for the discrepancies in fibrotic responses between the two TAC groups. PAT did not exert an evident effect on the LV abundance of myofibroblasts (green fluorescence, Fig. 6a), yet crucially increased LV expression of *Periostin* (*Postn*, Fig. 6b), which has previously been identified as most reliable marker for cardiac fibroblast activation [26]. Our data suggest that *Postn* is a crucial driver of PAT-mediated fibrotic responses upon pressure-overload, paving the way for the consequent progressive dysfunction [26]. Of note, while increased at HFrEF in the heart, *Postn* was decreased in the PAT by chronic pressure-overload (Supplementary Information Fig. S7). Regarding macrophages, PAT was not associated with expansion of overall LV CD68^+^ macrophages or herein of CD206^−^ or CD206^+^ alternatively activated/pro-fibrotic macrophages at the HFrEF stage, as assessed via flow cytometry and histology (Fig. 6c, d). Yet interestingly initial PAT removal clearly restricted peri-coronary accumulation of CD206^+^ (blue-lilac fluorescence) but not pro-inflammatory CD206^−^ (pink-red fluorescence) macrophages (Fig. 6e, f). Therefore, we speculate that in the presence of PAT, *Postn* promotes CD206^+^ macrophage accumulation around the arteries, as already described in pulmonary hypertension [67].

### Impact of PAT on LV fibroblast phenotype

**Fig. 7:**
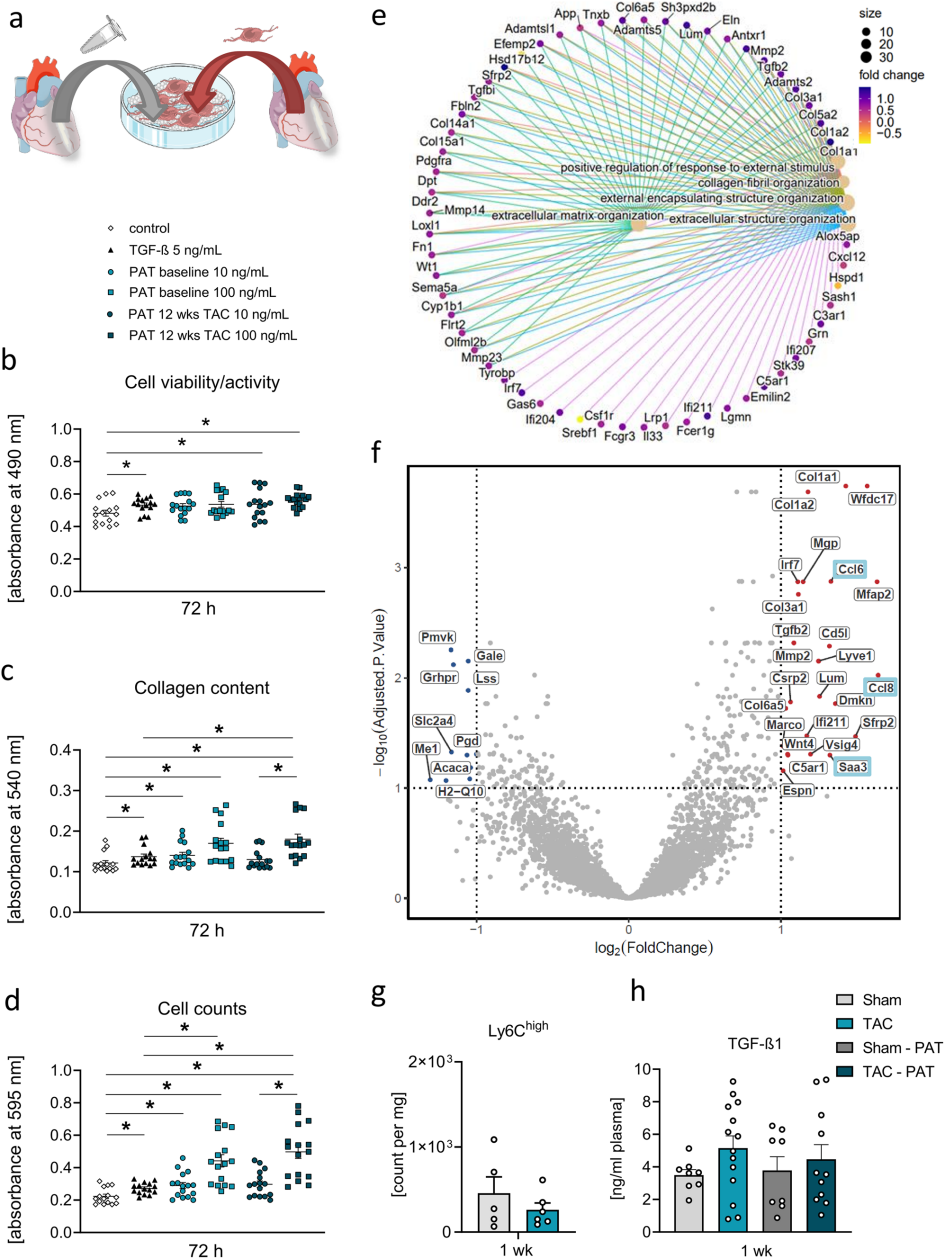
Impact of PAT on LV fibroblasts and of 1-week TAC on PAT transcriptional program. **a** Experimental design to assess impact of PAT proteins on cFB proliferation and activity. **b** Activation status, measured via MTT assay, **c** cell counts assessed via crystal violet staining and **d** collagen content measured via Sirius red staining following 72-h exposure to control (starvation media), TGF-β (5 ng/ml), or PAT protein (10 ng/ml; 100 ng/ml) from naïve or 12-week TAC mice. *n* = 3 independent cFB isolations with 3–6 wells per stimulation condition and isolation. Individual data are presented with mean ± SE. Kruskal–Wallis test with uncorrected Dunn’s post hoc test; *p < 0.05. **e** Cnet plot displaying significantly upregulated DEGs in PAT 1 week post-TAC vs. 1 week sham and annotated cellular components and **f** Volcano plot displaying unannotated significantly upregulated (red) and downregulated genes (blue) in PAT 1 week post-TAC vs. 1 week sham (*n* = 6/group) determined via bulk RNAsequencing. **g** Ly6C.^high^ monocytes per mg PAT 1 week post-intervention (*n* = 5–6/group). Bars indicate mean ± SE. **h** TGF-β1 plasma level assessed via ELISA 1 week post-intervention (*n* = 8–14/group). Two-way ANOVA with Sidak’s post hoc test; *p < 0.05

To further confirm a direct effect of PAT on the LV fibrotic response, we employed an in vitro approach in which cFBs from adult male WTs were exposed to PAT protein, extracted from naïve mice, and mice subjected to 12-week TAC, and subsequently characterized based on their activity and proliferation status (Fig. 7a). PAT protein lysate from 12-week TAC promoted cell viability and metabolic activity to a similar extent as TGF-β at both concentrations administered (Fig. 7b). Moreover, elevated cFB counts achieved by TGF-β could be mimicked by 10 ng/ml PAT protein, while 100 ng/ml PAT protein even significantly increased cell counts and also cFB collagen content compared to TGF-β, however independent of the PAT protein origin, i.e., naïve or 12-week TAC mice (Fig. 7c, d). These data provide evidence that PAT generated proteins can directly activate naïve cardiac fibroblasts. We speculate that the lack of an additional TAC effect in this in vitro approach can be explained by the fact that fibrotic responses in our in vivo study involve stressed fibroblasts exposed to, and already activated by, the pressure-overload as well as their concomitant, reciprocal interaction with stress-responding macrophages, and cardiomyocytes.

### Impact of LV pressure-overload on PAT transcriptional program

Next, we addressed our speculation that PAT exhibits fibroblast activating effects per se while the fat depot initiates a proper pro-fibrotic program only when interacting with the pressure-overloaded LV. We, therefore, performed PAT bulk RNAseq analyses 1 and 12 weeks post-surgery (Fig. 7e, f and Supplementary Information Figs. S7 and S8). Early pressure-overload (1 week) resulted in around 300 differentially regulated genes (DEG) in the PAT compared to 1-week sham PAT. Among those, 198 genes were significantly upregulated upon TAC vs. sham and, by vast majority, encoding ECM organizing and synthesizing factors, and herein various collagens (such as *Col1α1/2, Col3α1, Col5α2, Col14α1*), *Tgf-β2*, metalloproteases (such as *Mmp-2, -23, -14, Adamts-2, -5*), fibronectin 1, but also macrophage activation factors (such as *Csf1r, Il33, C5ar1*; Fig. 7e and Supplementary Information Fig. S8a). Moreover, we found that some of the most strikingly upregulated genes in the PAT upon TAC vs. sham are not annotated to the ECM but monocyte attraction and macrophage attraction/polarization such as *Ccl6* and *Ccl8,* but also serum amyloid A3 (*Saa3*, Fig. 7f, framed in light blue). These factors could be responsible for the more pronounced recruitment of Ly6C^high^ monocytes and macrophages to the myocardium 1 week post-TAC in the presence of the adjacent intact PAT. Flow cytometry PAT data further support the hypothesis that PAT rather represents a source of mediators than of monocytes recruited to the heart as shown by widely unchanged PAT Ly6C^high^ monocyte counts between sham and TAC conditions at a time point when we had observed increased Ly6C^high^ infiltration into the myocardium in the presence of PAT upon TAC (Fig. 7g). These data indicate that PAT itself senses the mechanical load exposed on the heart and induces a monocyte/macrophage recruiting and an ECM synthesizing and organizing transcriptional program. Based on our observations, we may speculate that this local response results in the release of chemo-attractants and pro-fibrotic factors which, in turn,modulate cardiac fibroblast and macrophage responses. This, however, seem not to predominantly include promoted secretion of TGF-β1 from the PAT/in the presence of PAT (Fig. 7h). While gaining a more ECM- and immune-modulatory character, PAT loses its predominant function as a metabolic tissue, providing especially fatty acids, in response to TAC, as shown by downregulated genes allocated especially to cellular respiration and fatty acid oxidation (Supplementary Information Fig. S8b).

At the HFrEF stage, 12 weeks post-TAC, we only found 41 PAT genes upregulated compared to 1-week TAC, suggesting that the majority of pressure-overload-induced changes in the PAT transcriptome occurs as an early response paving the way for long-term effects in the underlying myocardium. Apart from some distinctly regulated genes such as angiotensinogen (*Atg*), which could contribute to a vasoconstriction upon translation and release (Supplementary Information Fig. S7), the majority of upregulated genes due to TAC duration mainly encode for leukocyte and herein lymphocyte activation (Supplementary Information Fig. S9a). This activation was not associated with PAT lymphocyte expansion (Supplementary Information Fig. S9b). We also found rather a general trend toward lower cardiac lymphocyte counts in 12-week TAC vs. 12-week sham mice independent of PAT presence which, however, seemed not to derive from time-dependent decrease following TAC yet, rather from time-dependent expansion under sham conditions (Supplementary Information Fig. S10). Circulating and splenic T cells, in contrast, hint toward a decrease in numbers at the advanced disease stages independent of PAT (Supplementary Information Figs. S10–11) with the latter especially in the TAC group associated with decreased *Atg1r* transcription (Supplementary Information Fig. S11e). AT1 receptor has been shown in vitro to mediate proliferation of murine splenic lymphocytes and is known to regulate T-cell expansion, differentiation, and function [71]. The observed alteration in lymphocyte counts under long-term pressure-overload needs to be elaborated by future research yet, seems not to causally contribute to the functional outcome in our study since it was unaffected by PAT presence. Of note, PAT removal per se was associated with a time-dependent increase in splenic lymphocyte numbers which might result from the lack of homing capability to the tertiary lymphocyte depot, the PAT.

## Discussion

Our data demonstrate that PAT does not solely contribute to pressure-induced HF secondary to its own metabolic remodeling, and mass increase due to systemic metabolic disorders. We rather revealed that the fat depot adversely impacts LV remodeling and function even though its mass did not correlate with HFrEF severity, which is in accordance with some clinical EAT data [28, 60]. A reduction of (not previously expanded) EAT volume/mass during HFrEF due to empagliflozin treatment, however, still limits remodeling and improves function in non-diabetic patients [49]. We could confirm a similar beneficial effect for the removal of PAT by demonstrating that the surgical excision prevented progressive transition into an eccentric dilated HFrEF phenotype between 8 and 12 weeks of pressure-overload by maintaining concentric hypertrophy. This observation hints toward a so far unknown role for PAT in mechanically induced HF progression and could, therefore, also be of high relevance for prognosis and management of patients with aortic stenosis. The protection against progressive deterioration included restriction of *Μyh7* upregulation and a timely resolved LV re-expression of *Nppa* and *Nppb* post-TAC. The latter implies that PAT increases LV filling pressure, as further underpinned by progressive increase in LV mass and lung congestion, hinting toward more pronounced diastolic impairment, which was counteracted by initial PAT removal [13, 17, 19, 31].

Deterioration of systolic function in turn is reflected by myocardial noradrenaline accumulation and vice versa HF is associated with sympathetic hyperactivity in the EAT in the absence of its hyper-innervation [21, 28]. The latter concurs with our findings in the murine PAT during HF. Herein, we could not find increased TH fiber length or PAT impact on plasma NE level (potentially released from the PAT to enter the heart) or LV *Adrb1* expression during advanced disease stages.

Cardiac fat depots have long been mainly suggested as energy providers for the heart yet, adipocytes and adipocyte-derived stem cells gain increasing perception as mechano-sensitive and mechano-responsive cells, which release pro-inflammatory and -fibrotic molecules in response to LV remodeling and the associated abnormal mechanical stress [29, 72]. Accordingly in vitro experiments have shown that mechano-transduction contributes to fibro-inflammation in human adipocytes [44]. Our study suggests a similar mechanism in the murine PAT to occur following TAC, and further to be responsible for the transition from adverse remodeling to HFrEF upon long-term pressure-overload. Local changes towards a more pro-fibrotic and pro-inflammatory milieu impact the diseased myocardium, potentially directly by molecules traveling via pericardial pores or indirectly via release into the circulation [39]. Different cardiac AT depots exhibit distinct proteomic and transcriptomic signatures with differential effects on the adjacent cardiac structures [21]. We revealed that PAT initiates a transcriptional program encoding for ECM deposition, and BMDM recruiting factors as well as macrophage activation while genes involved in fatty acid metabolism were downregulated early after initiation of LV wall stress. A direct transcriptional cardiac AT response to cardiac stress has already been reported by clinical studies. In this context, advanced coronary artery disease was associated with whitening, fibrosis, and apoptosis of EAT, characterized by downregulation of pro-thermogenic genes and a concomitant upregulation of pro-inflammatory cytokine transcription [36, 52]. Moreover, HF patients exhibit elevated MMP-14 level and an upregulated mechano-sensitive IL-33-inducing cascade, correlating with maladaption such as LV enlargement, while 1-week TAC increased *Mmp-14* and *Il-33* transcription in PAT in our mouse model, suggesting MMP-14 and IL-33 as potential (early) AT marker for HF development [58, 73].

We might also speculate that PAT, sensing the mechanical load on the heart, subsequently exerts mechanical LV wall stretch itself, thereby promoting the observed eccentric cardiac remodeling in our study. The mechanical impact of PAT (and most likely EAT in lean objects) on the pressure-overloaded LV differs from the mechanical disturbance generated by obesity-driven EAT accumulation. In obese HFpEF patients, EAT expands in the parietal sac, thereby exerting pericardial restraint and myocardial compression with the consequent restriction of filling reflected by increased E/A ratio, reduced deceleration time, and shortened isovolumetric relaxation time, while EAT mass reduction unloads the heart, alleviating the described pathology [5, 7, 28, 54, 57, 66]. In contrast, PAT is not expanding during HFrEF and, beyond this, PAT is not located in the pericardial sac but attached to the fibrous pericardium and therefore not associated with the described MV flow characteristics. Of note, a partly resection of the pericardium is inevitable when surgically excising the attached PAT. Yet, under unstressed sham conditions, LV filling pressure markers were unaffected by the PAT removal surgery, and moreover neither sham nor TAC cohorts exhibited MV flow pattern indicative for compression or restricted filling pathology compared to their -PAT counterparts [5, 7]. Consequently, PAT does not exert pericardial restraint as described above. We rather suggest the mechanical effect of PAT to derive from its own fibrotic remodeling resulting in increased stiffness and a longitudinal stretch but not circumferential compression, thereby contributing to the eccentric dilation in the long term upon pressure-overload which is absent in the TAC-PAT cohort.

Cellular transcriptomic changes 1 week after pressure-overload exposure by TAC seem not only to be transient but also sufficient to pave the way for fibrotic remodeling in the long term. Such timely and transient regulations leading subsequently to LV fibrosis have also been revealed by bulk and single-cell RNAsequencing in endothelial cells and fibroblasts at 1-week TAC compared to 1-week sham and to 8-week TAC in male BL/6N mice [16]. Concurring with the hypothesis of early changes to be determinant for the outcome, we could not find prominent PAT transcriptome changes between 1 and 12 weeks post-TAC.

In turn, we detected marked *Postn*, *Il-6*, *Col3α1*, *Ctgf* expression (correlating with the HF marker *Nppb*), dilation, systolic deterioration, and lung congestion at the 12-week time point which could be prevented by PAT removal. This outcome is similar to the effects of CTGF inhibition during maladaptive pressure-induced LV remodeling and HF in mice [55]. Moreover, while the relevance of α-SMA as the major marker for LV pressure-activated fibroblasts has been challenged recently, single cardiac fibroblast sequencing has identified *Postn* as the most pronounced, mechanical load-induced marker for activated fibroblasts, also strongly correlating with *Ctgf*, and a *Postn* ablation has been shown to counteract pressure-induced (AngiotensinII) maladaptive remodeling and EF reduction in mice [26, 37]. These data again support the hypothesis of a PAT-mediated mechanical LV stretch and suggest a direct pro-fibrotic effect of PAT. However, myocardial fibrosis is achieved by complementary, reciprocal fibroblast–macrophage interactions. Fibroblasts can recruit macrophages to fibrotic areas and direct them toward an alternatively activated phenotype [65]. In our study, excessive perivascular fibrosis in 12-week TAC mice was accompanied by peri-coronary accumulation of pro-fibrotic CD206 + macrophages. In agreement with these findings, a histological study reports 4-week TAC mediated fibrosis to predominantly localize in the perivascular area in the murine heart. This was preceded by accumulation of Galactin-3 positive cells, which can express CD206, as shown for example in liver fibrosis and post-MI wound healing in mice [6, 63]. In turn, not only fibroblasts can activate macrophages but also vice versa BMDMs aggravate fibrotic remodeling by activating fibroblasts, an interaction which crucially involves IL-1β signaling, as recently shown [2, 3, 50, 59, 70]. Our data show that PAT initiates a macrophage activation program while simultaneously promoting early pro-inflammatory BMDM recruitment to the heart, likely via secretion of CCL6, CCL8, and SAA3, as suggested by their high PAT mRNA expression levels, and LV *Il-1β* expression, implying that PAT thereby amplifies innate immune responses and fibrotic remodeling, contributing to HFrEF establishment [8]. Of note, while CCL8-SAA3 are involved in recruitment of pro-inflammatory monocytes, CCL6 has been shown to inhibit autophagy and to polarize macrophages to alternatively activated CD206 expressing subsets during wound healing [15]. Moreover, PAT-mediated LV upregulation of *Postn* is likely to promote CD206^+^ macrophage accumulation around the arteries, as already described for pulmonary hypertension [67]. These observations could, therefore, explain the peri-coronary CD206 + subset accumulation in the pressure-overloaded heart in the presence of PAT. Our flow cytometry PAT data further support the hypothesis that PAT rather represents a source of mediators than of myeloid cells recruited to the heart. BMDM recruitment, in turn, could also be restricted by reduced monocyte egress from hematopoietic stores in the absence of PAT. While further studies are warranted to unravel the factors and mechanisms by which PAT exerts fibrotic responses, we currently speculate these to derive from both, direct and BMDM-mediated fibroblast activation. Fibrosis is a key detrimental player in pressure-overload-induced HF, disturbing myocardial compliance, perfusion and excitation–contraction coupling, therefore representing a process sufficient per se to determine extent of diastolic and systolic functional deterioration. EAT remodeling during coronary artery disease and atrial fibrillation is associated not only with cardiac fibrosis but also with microvascular dysfunction and rarefication [23, 32, 40, 61, 64]. Coronary vasoconstriction, endothelial dysfunction, and increased extravascular coronary compression are general hallmarks of and contributors to HF [18]. In our TAC model where pressure is also exerted on the coronary circulation, we also hypothesize a direct effect of PAT on microvascular function. On the one hand, we suggest a direct link between perivascular fibrosis driven by PAT and arterial and microvascular impairment. This could be achieved via collagen deposition-driven perivascular compression, in combination with increased diastolic filling pressure, as suggested by promoted *Nppa* and *Nppb* expression and lung congestion in the presence of PAT, but also by upregulation of *Agt* in the PAT during HFrEF. The latter could further contribute, as a secreted neurohumoral factor, to vasoconstriction, eventually aggravating systolic deterioration [18, 35]. This, however, needs to be confirmed by further mechanistic studies. On the other hand, we speculate the PAT-mediated inflammatory response including release of soluble (inflammatory and vasoconstrictive) substances, potentiated LV macrophage expansion, and LV upregulation of *Il-1β* and *Il-6* to further contribute to coronary microvascular impairment and thereby to the observed systolic deterioration [18, 21, 27]. Besides, it is quite conceivable that in HFpEF patients, PAT expansion aggravates coronary dysfunction even further via potentiation of systemic inflammation and changes in metabolic substrate provision for the heart.

## Conclusion

Our study revealed for the first time a contributory role of PAT to the progression of HF, independent of metabolic impairment, by demonstrating that PAT removal counteracts purely pressure-overload-mediated manifestation of HFrEF in male mice. This protection seems to mainly derive from attenuation of LV stretch and fibrotic responses which encompasses prevention of pro-fibrotic gene expression, fibroblast activation, and alleviation of early pro-inflammatory and late pro-fibrotic, pericoronary macrophage accumulation in the heart. We speculate these benefits to derive from the lack of secreted factors from the PAT after its removal, as suggested by its pressure-induced transcriptional program. Restricting the availability of the harmful PAT secretome to the heart could, therefore, represent a promising strategy to attenuate adverse remodeling and concomitant HF progression even in the absence of metabolic impairment. We believe our insights to pave the way for subsequent studies further elucidating targetable underlying mechanisms.

### Study limitations

Our study comprehensively investigated the impact of surgical PAT removal on pressure-induced HF progression and led to the identification of inflammatory and fibrotic processes modulated by PAT but also unraveled modulations of the PAT itself in response to LV afterload. Nonetheless, future mechanistic studies are warranted to elaborate the underlying mechanisms by which PAT exerts its effects on the pressure-overloaded heart.

## Supplementary Information

Below is the link to the electronic supplementary material.

Supplementary file1 (DOCX 1559 KB)

## Data Availability

The RNAsequencing data underlying this article are available in an open access repository (GEO, series: GSE283668) and are accessible via the following link https://www.ncbi.nlm.nih.gov/geo/query/acc.cgi?acc=GSE283668. All other data underlying this article are available in the article and in its online supplementary information.

## Declarations

### Software

The graphical abstract has been created with BioRender.
