# Supplementary material for: Pericardial adipose tissue promotes transition to heart failure with reduced ejection fraction upon pressure-overload in mice
Source: Basic Res Cardiol. 2025 Jul 3;120(5):925–45. doi: 10.1007/s00395-025-01116-x (PMC12518406; doi:10.1007/s00395-025-01116-x)
Supplement: Supplementary file 1 — Supplementary file1 (DOCX 1559 KB) [file 395_2025_1116_MOESM1_ESM.docx]

**Supplementary Information**

**Pericardial adipose tissue promotes transition to heart failure with reduced ejection fraction upon pressure-overload in mice**

Yi Xuan Shia^1^, Kathleen Pappritz^3,4^, Anna Cristina Kaltenbach^1^, Guo Li^1^, Valentina Fardella^5^, Sophie Van Linthout^3,4^, Daniela Carnevale^5,6^, Sabine Steffens^1,7^, Sarah-Lena Puhl^1,2^

^1^Institute for Cardiovascular Prevention (IPEK), Ludwig-Maximilians-Universität (LMU) Munich, Munich, Germany

^2^Comprehensive Heart Failure Center, University Clinic Würzburg, Würzburg, Germany

^3^Berlin Institute of Health at Charité, Universitätsmedizin Berlin, BIH Center for Regenerative Therapies (BCRT), Berlin, Germany

^4^German Center for Cardiovascular Research (DZHK), partner site Berlin, Berlin, Germany

^5^Research Unit of Neuro and Cardiovascular Pathophysiology, IRCCS Neuromed, Pozzilli, Italy

^6^Department of Medical and Surgical Sciences and Biotechnologies, "Sapienza" University of Rome, Rome, Italy

^7^German Center for Cardiovascular Research (DZHK), partner site Munich Heart Alliance, Munich, Germany

Correspondence: Sarah-Lena Puhl; [puhl_s@ukw.de](mailto:puhl_s@ukw.de); Comprehensive Heart Failure Center, University Clinic Würzburg, Am Schwarzenberg 15, 97078 Würzburg, Germany; Phone: +49 931 201-46463

**Content**

-Supplementary Figures

-Supplementary Tables

-Supplementary Methods

**Supplementary Figures**

**
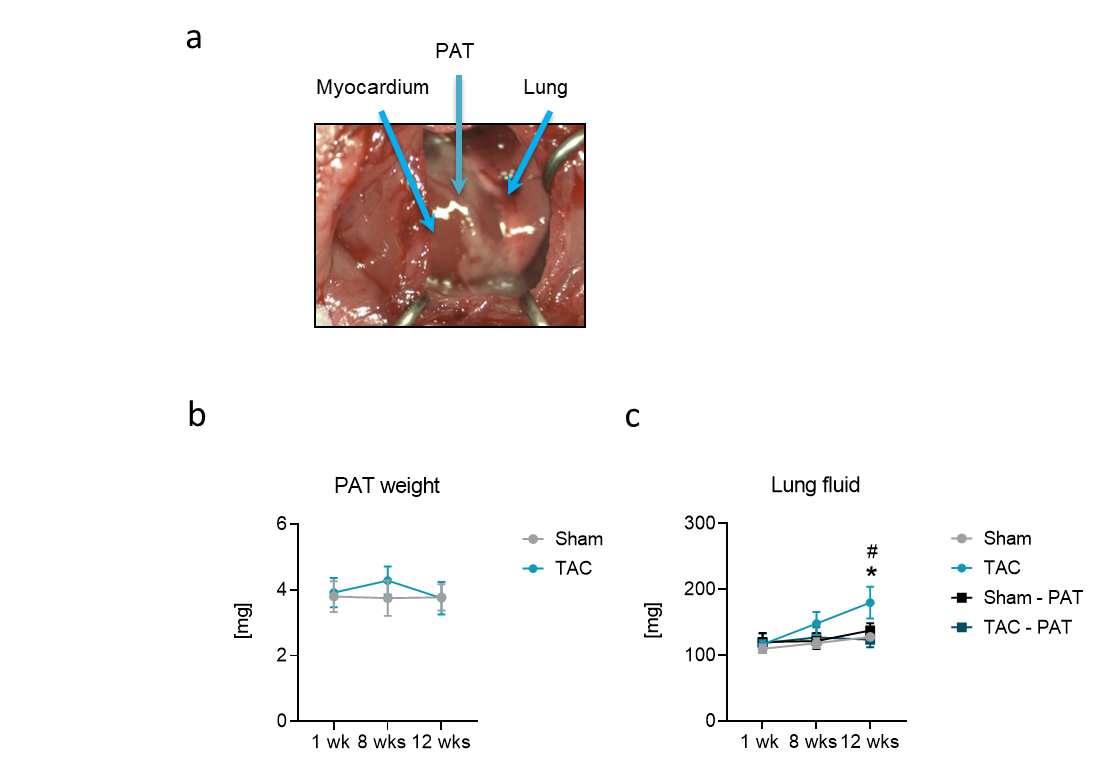
**

**Fig. S1 Murine PAT and impact of TAC duration on PAT weight and lung fluid**

a) Photograph of an open murine chest displaying the myocardium, the left lung and the PAT (white fat string attached to the myocardium) which has been surgically excised in the Sham – PAT and TAC – PAT groups of the study. b) PAT weight 1, 8 and 12 wks post intervention (n=7-14/group). c) Lung fluid weight 1, 8 and 12 wks post intervention (n=6-14/group). Symbols indicate mean±SE; two-way ANOVA with Tukey’s post hoc test; p<0.05; * vs. Sham; # vs. TAC - PAT.

**
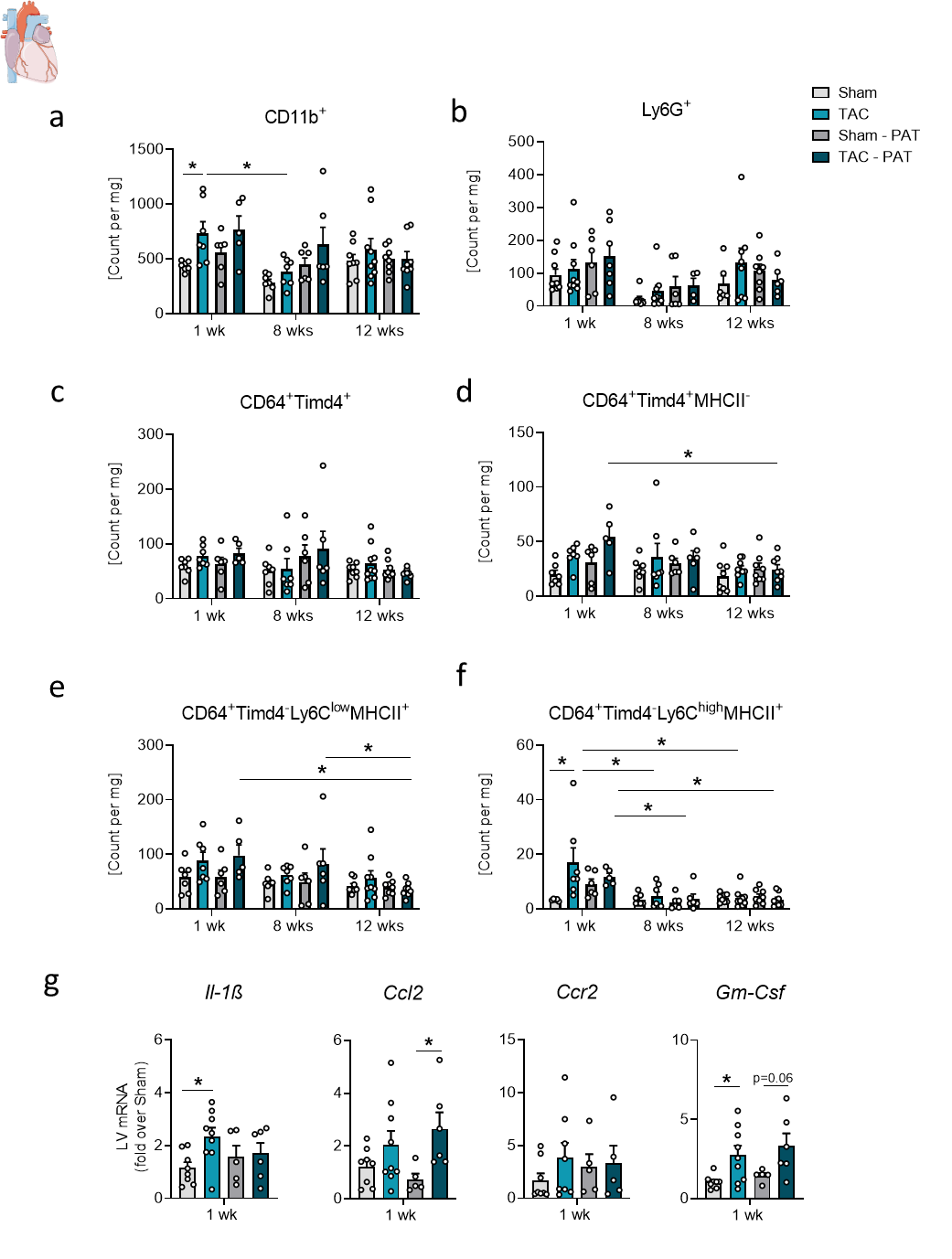
**

**Fig.** **S2** **Impact of PAT removal on pressure-overload associated LV inflammation**

Counts of a) all CD11b^+^ myeloids, b) Ly6G+ neutrophils, c) resident Timd4^+^ macrophages and herein d) phagocytic MHCII^-^ subsets, e) recruited, reparative Ly6C^low^MHCII^-^ macrophages and f) recruited pro-inflammatory antigen-presenting Ly6C^high^MHCII^+^ macrophages per mg heart 1 wk (n=5-7/group), 8 wks (n=6-9/group) and 12 wks (n=8-10/group) post-intervention. Bars indicate mean±SE; two-way ANOVA with Tukey’s post hoc test. g) LV mRNA expression of pro-inflammatory *Il-1β* and *Ccl2*, *Ccr2* and *GM-Csf1* 1 wk post-intervention relative to Sham (n=5-9/group). Bars indicate mean±SE; two-way ANOVA with Sidak’s post hoc test; *p<0.05.


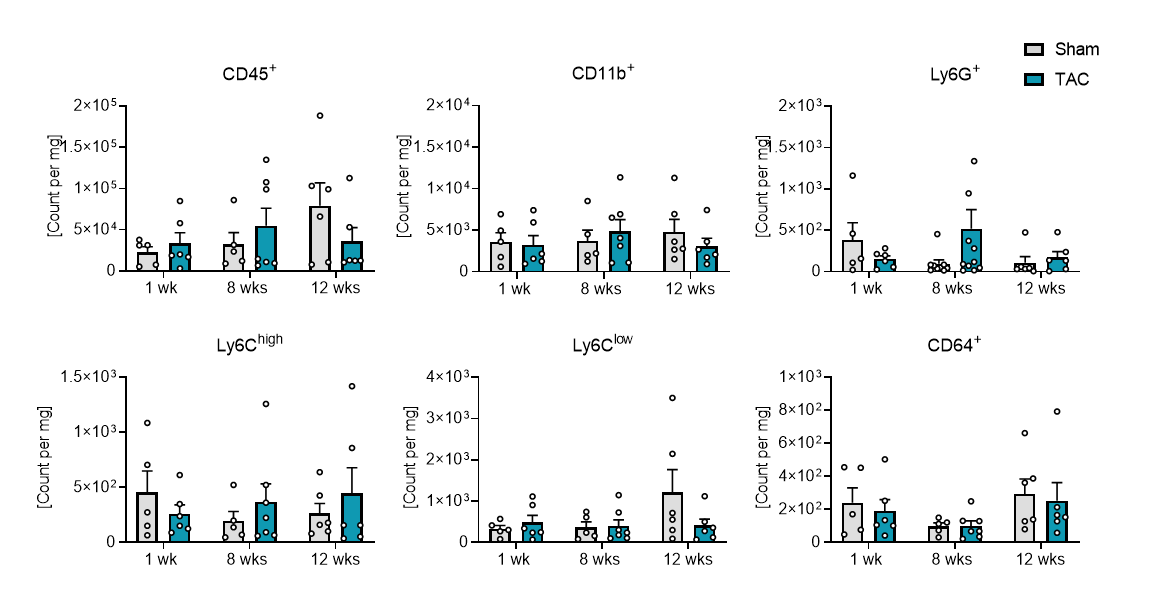


**Fig. S3** **Impact of pressure-overload on PAT myeloid cell composition**

Counts of all CD45^+^ leukocytes, CD11b^+^ myeloids, Ly6G+ neutrophils, Ly6C^high^ and Ly6C^low^ monocytes and macrophages per mg PAT 1, 8 and 12 wks post-intervention (n=5-7/group). Bars indicate mean±SE.

**
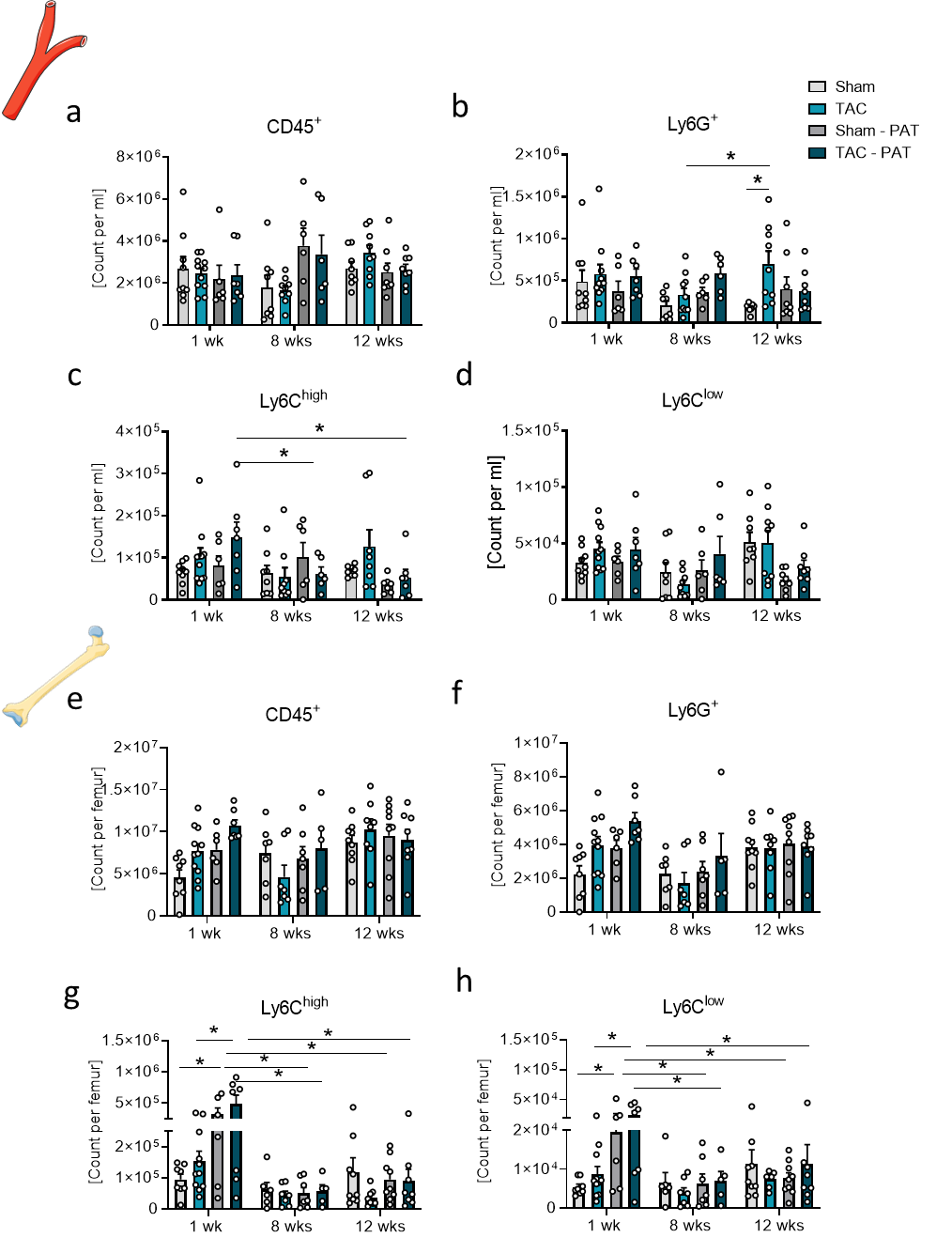
**

**Fig. S4** **Impact of PAT removal on circulating and femoral leukocyte counts upon pressure-overload**

Counts of a+e) all CD45^+^ leukocytes, b+f) Ly6G^+^ neutrophils, c+g) Ly6C^high^ and d+h) Ly6C^low^ monocytes per ml blood (upper rows, n=6-11/group) and per femur (bottom rows, n=5-11/group), respectively, 1, 8 and 12 wks post-intervention (n=5-13/group). Bars indicate mean±SE; two-way ANOVA with Tukey’s post hoc test; *p<0.05.


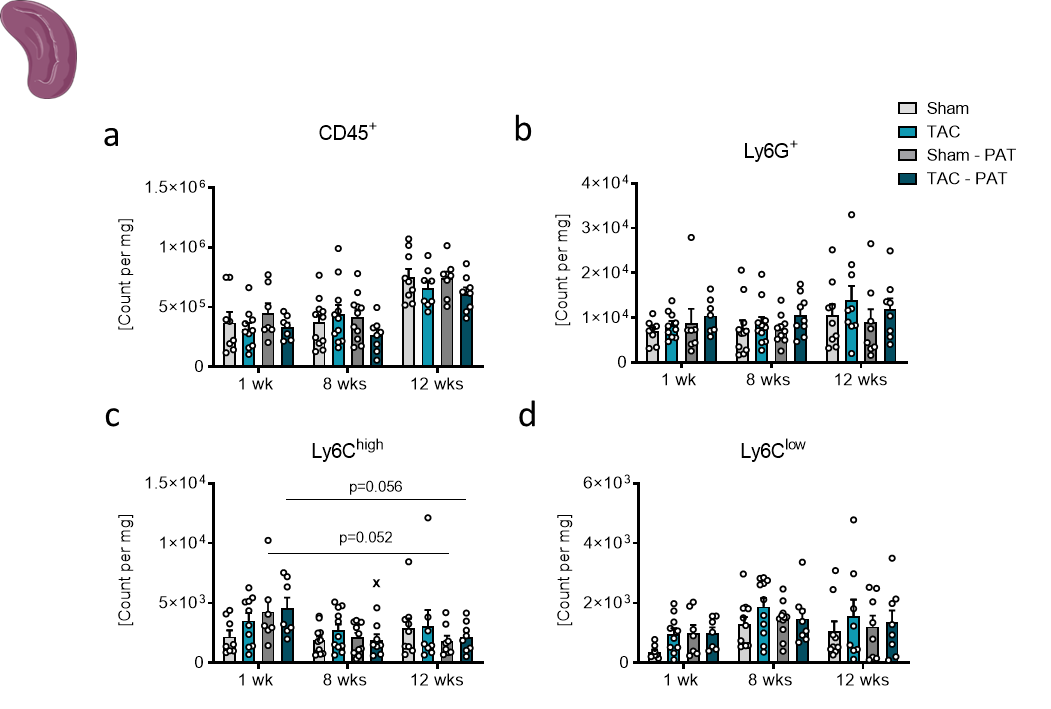


**Fig. S5** **Impact of PAT removal on splenic leukocyte counts upon pressure-overload**

Counts of a) all CD45^+^ leukocytes, b) Ly6G^+^ neutrophils, c) Ly6C^high^ and d) Ly6C^low^ monocytes per mg spleen 1, 8 and 12 wks post-intervention (n=8-11/group). Bars indicate mean±SE; two-way ANOVA with Tukey’s post hoc test; * p<0.05.


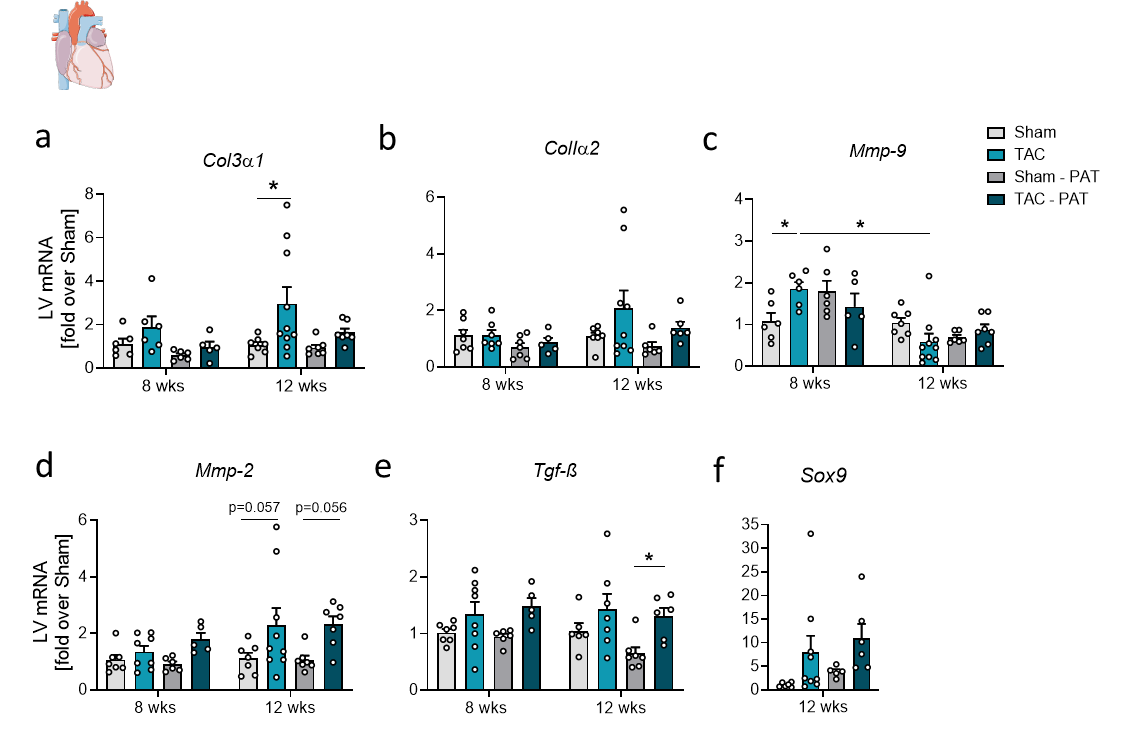


**Fig. S6 Impact of PAT removal on pro-fibrotic gene expression upon pressure-overload**

LV mRNA expression of a) *Col3α1*, b) *Col1α2*, c) *Mmp-9,* d) *Mmp-2*, e) *Tgf-β* 8 and 12 wks post-intervention and f) *Sox9* 12 wks post-intervention relative to Sham data of the respective time-point (n=5-9/group). Bars indicate mean±SE; two-way ANOVA with Tukey’s post hoc test or Sidak’s post-hoc test, as appropriate; *p<0.05.

**Fig. S7 Impact of long-term pressure-overload on PAT transcriptional program**

Volcano plot displaying unannotated most significantly DEGs in PAT 12 wks post-TAC vs. 1 wk TAC (n=6-7/group) determined via bulk RNAsequencing.


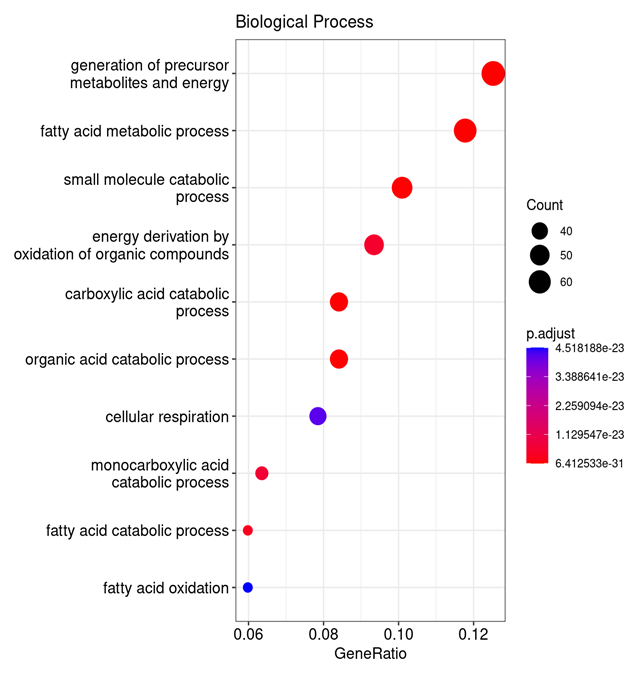

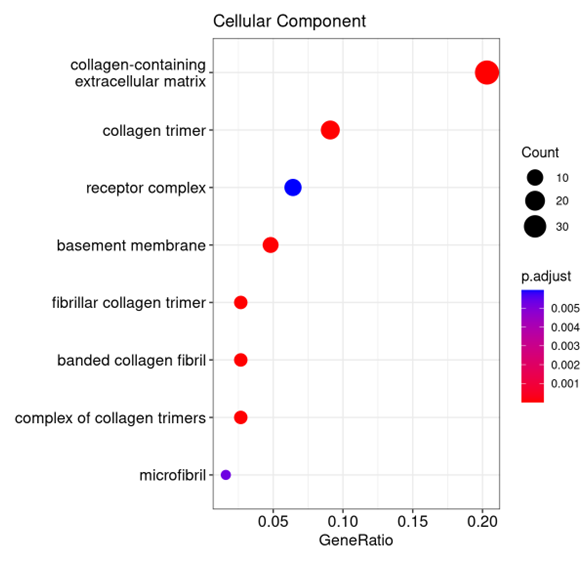


**b**

**a**

**Fig. S8 Impact of early pressure-overload on the PAT transcriptional program**

a) Cellular components annotated based on 198 differentially up-regulated genes (p<0.1) in PAT 1 wk post-TAC compared to 1 wk post-Sham (n=6/group). b) Biological processes annotated based on 102 differentially down-regulated genes (p<0.1) in PAT 1 wk post-TAC compared to 1 wk post-Sham.


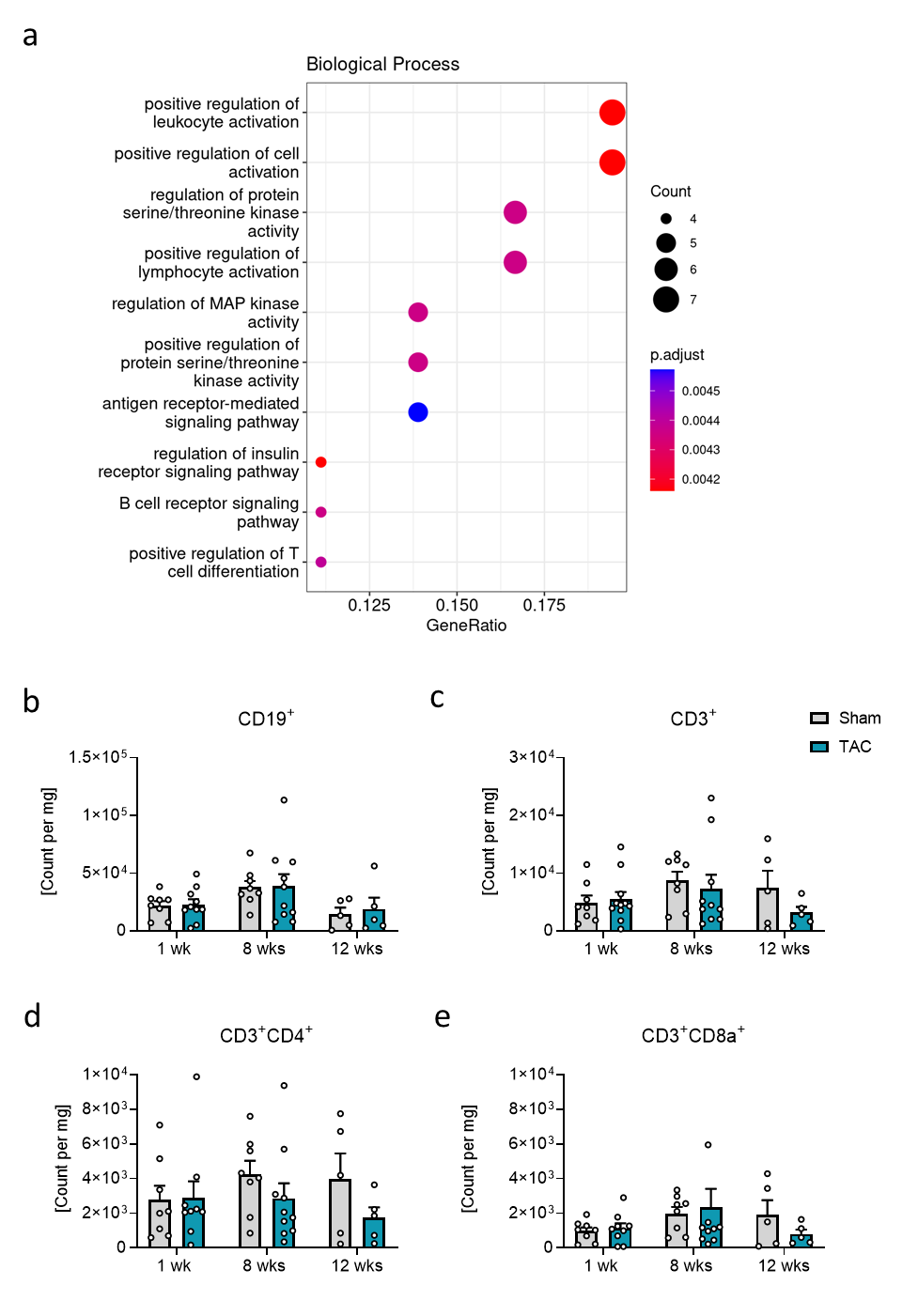


**Fig. S9 Impact of HFrEF on the PAT transcriptional program and PAT lymphocyte composition**

a) Biological processes annotated based on 41 differentially up-regulated genes (p<0.1) in PAT 12 wk post-TAC compared to 1 wk post-TAC (n=6-7/group). Counts of b) CD19^+^ B-cells, c) CD3^+^ T-cells and amongst those of d) CD4^+^ helper and e) CD8a^+^ cytotoxic subsets per mg PAT 1 wk (n=8-9/group), 8 wks (n=8-10/group) and 12 wks (n=5/group) post-intervention. Bars indicate mean±SE; two-way ANOVA with Sidak’s post hoc test.


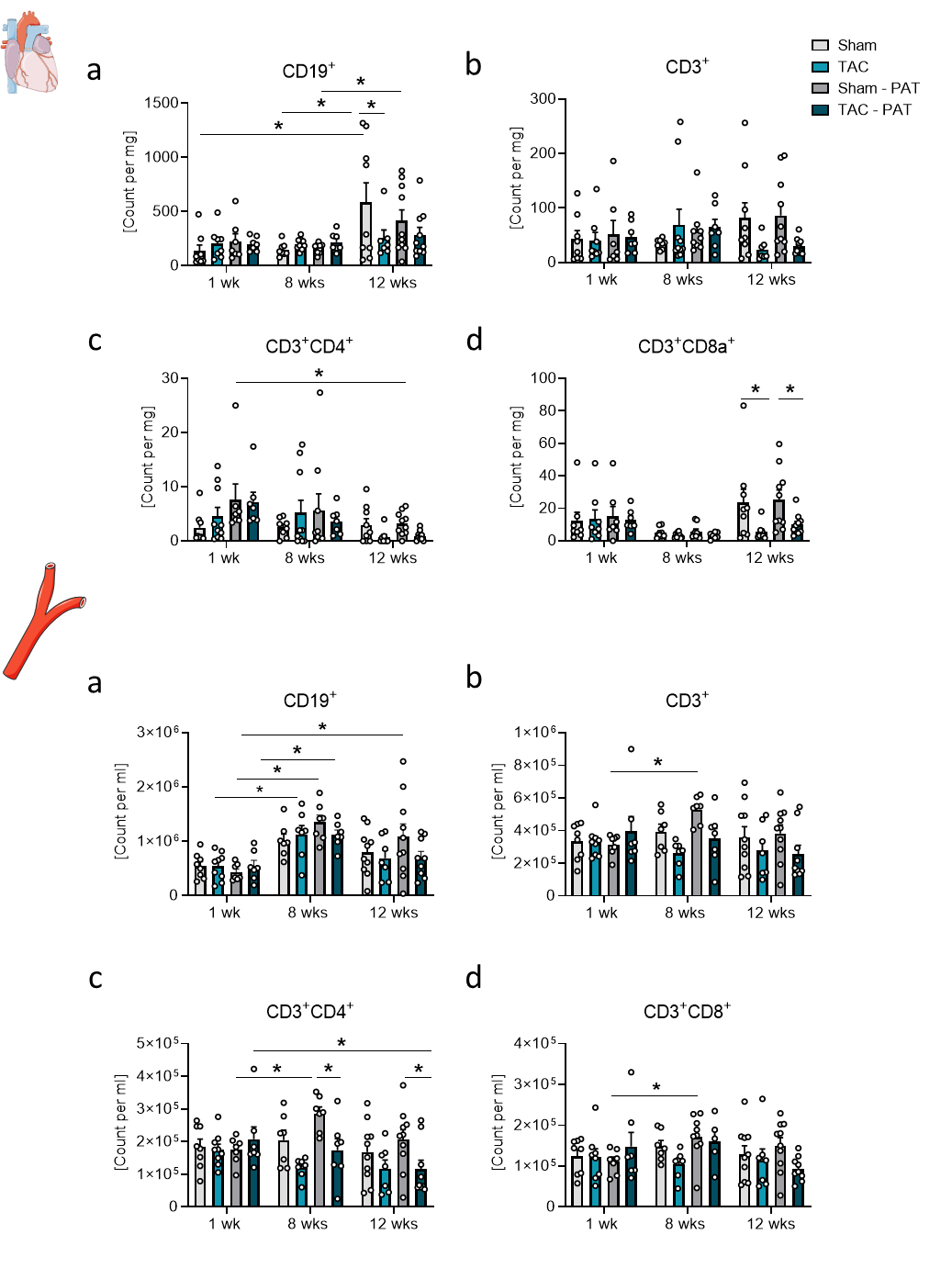


**Fig. S10 Impact of pressure-overload on cardiac and circulating lymphocyte counts**

a) CD19^+^ B-cells, b) CD3^+^ T-cells and amongst those of c) CD4^+^ helper and d) CD8a^+^ cytotoxic subsets per mg heart (upper rows; n=7-10/group) and per ml blood (bottom rows; n=7-10/group) 1, 8 and 12 wks post-intervention (n=7-10/group). Bars indicate mean±SE; two-way ANOVA with Tukey’s post hoc test; *p<0.05.


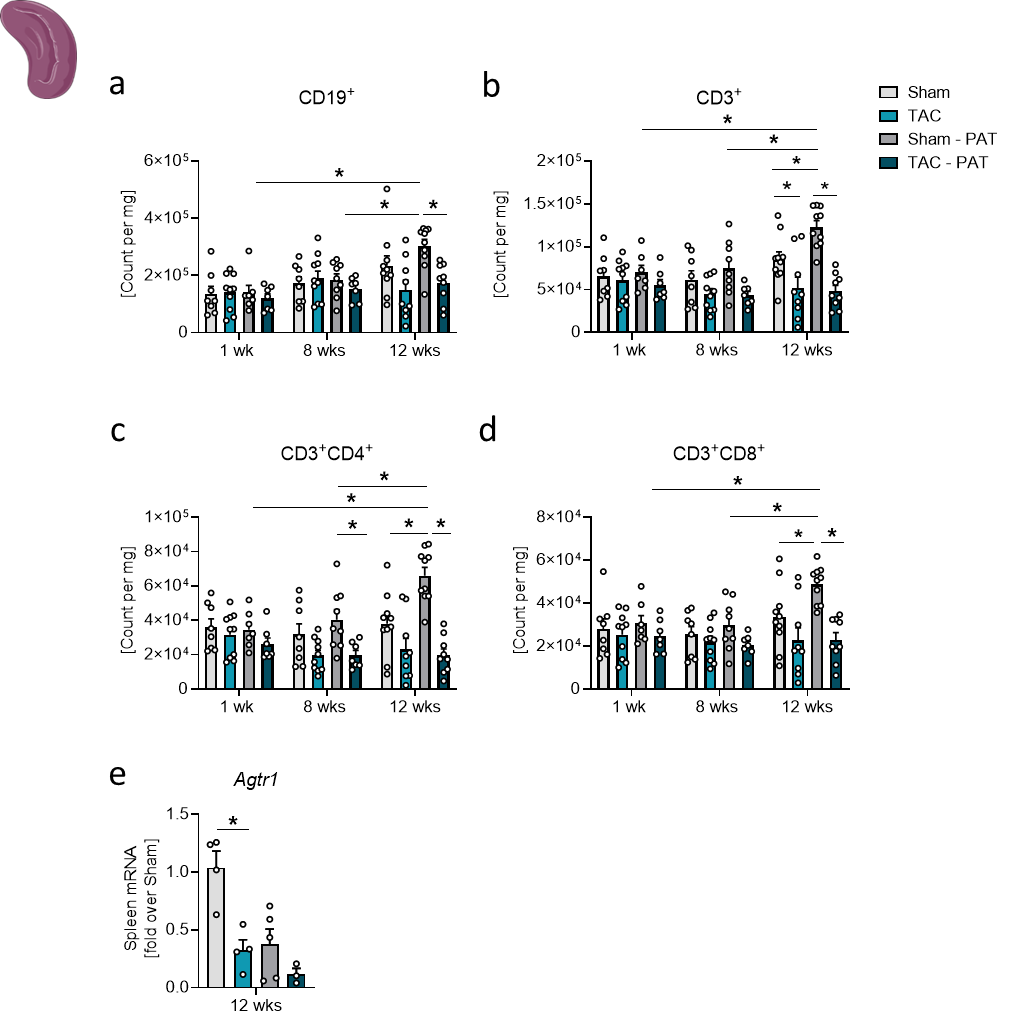


**Fig. S11 Impact of pressure-overload on splenic lymphocyte counts**

a) CD19^+^ B-cells, b) CD3^+^ T-cells and amongst those of c) CD4^+^ helper and d) CD8a^+^ cytotoxic subsets per mg spleen 1, 8 and 12 wks post-intervention (n=7-10/group). e) Splenic mRNA expression *Atgr1* 12 wks post-intervention relative to Sham data (n=3-5/group). Bars indicate mean±SE; two-way ANOVA with Tukey’s post hoc test or Sidak’s post-hoc test, as appropriate; *p<0.05.

**Supplementary Tables**

**Suppl. Table S1: Phenotype of male mice, aged 5 months, 12 weeks post-surgery determined via gravimetry**

|  | **Sham** | **TAC** | **Sham-PAT** | **TAC-PAT** |
| --- | --- | --- | --- | --- |
| ***Body dimensions*** |  |  |  |  |
| **n** | **10-18** | **10-18** | **10-14** | **9-12** |
| **Body weight [g]** | 31.2 ± 0.4 | 29.2 ± 0.9 | 29.4 ± 0.5 | 29.4 ± 0.7 |
| **Tibia length [mm]** | 18.3 ± 0.1 | 18.1 ± 0.1 | 18.2 ± 0.1 | 18.2 ± 0.1 |
| ***Heart chamber weights*** |  |  |  |  |
| **n** | **18** | **18** | **14** | **12** |
| **Whole heart [mg]** | 160.5 ± 4.4 | 261.6 ± 16.0 *# | 149.6 ± 4.9 | 214.4 ± 11.4 * |
| **Atria [mg]** | 9.7 ± 0.7 | 20.6 ± 4.2 * | 9.3 ± 0.5 | 13.7 ± 1.2 |
| **Ventricles [mg]** | 150.8 ± 4.3 | 240.9 ± 13.5 *# | 140.4 ± 4.8 | 200.8 ± 10.6 * |
| ***Wet organ weights*** |  |  |  |  |
| **n** | **10-18** | **9-18** | **10-14** | **9-12** |
| **Lungs [mg]** | 159.9 ± 4.8 | 261.7 ± 36.5 *# | 166.9 ± 10.9 | 153.5 ± 11.7 |
| **Liver [mg]** | 1449 ± 43 | 1392 ± 76 | 1419 ± 49 | 1455 ± 51 |
| **Kidney [mg]** | 185.7 ± 6.8 | 169.7 ± 6.4 | 173.1 ± 5.8 | 184.3 ± 9.5 |
| **Spleen [mg]** | 100.2 ± 6.8 | 92.1 ± 8.4 | 87.2 ± 4.8 | 106.0 ± 12.3 |
| ***Dry organ weights*** |  |  |  |  |
| **n** | **10-18** | **9-18** | **10-14** | **9-12** |
| **Lungs [mg]** | 31.8 ± 1.2 | 50.2 ± 6.3 *# | 29.1 ± 0.8 | 32.1 ± 2.7 |
| **Liver [mg]** | 467 ± 15 | 446 ± 23 | 464 ± 20 | 455 ± 14 |
| **Kidney [mg]** | 49.8 ± 1.7 | 43.3 ± 1.7 * | 45.6 ± 1.7 | 47.8 ± 2.7 |
| ***Wet organ weights normalized to body weight [mg/g]*** | | |  |  |
| **n** | **18** | **11-18** | **14** | **12** |
| **Heart [mg]** | 4.8 ± 0.1 | 8.3 ± 0.6 *# | 4.8 ± 0.1 | 6.9 ± 0.4 * |
| **Spleen [mg]** | 3.2 ± 0.2 | 3.5 ± 0.4 | 3.0 ± 0.1 | 3.6 ± 0.4 |
| ***Wet organ weights normalized to tibia length [mg/mm]*** | | |  |  |
| **n** | **10** | **10** | **10** | **9** |
| **Heart [mg]** | 8.3 ± 0.4 | 12.5 ± 0.9 * | 7.4 ± 0.3 | 11.7 ± 0.6 * |
| **Spleen [mg]** | 6.1 ± 0.6 | 5.8 ± 0.9 | 4.5 ± 0.2 | 6.0 ± 0.8 |

Mean ± SE, *p<0.05 vs. corresponding Sham, #p<0.05 vs. TAC; Two-way ANOVA with Sidak’s post hoc test

**Suppl. Table S2: Phenotype of male mice, aged 4 months, 8 weeks post-surgery determined via gravimetry**

|  | **Sham** | **TAC** | **Sham-PAT** | **TAC-PAT** |
| --- | --- | --- | --- | --- |
| ***Body dimensions*** |  |  |  |  |
| **n** | **11-12** | **11-15** | **11-13** | **9-12** |
| **Body weight [g]** | 29.3 ± 0.3 | 28.6 ± 0.6 | 27.7 ± 0.5 | 28.0 ± 0.8 |
| **Tibia length [mm]** | 18.1 ± 0.1 | 18.1 ± 0.1 | 18.2 ± 0.1 | 18.2 ± 0.1 |
| ***Heart chamber weights*** |  |  |  |  |
| **n** | **11** | **15** | **12** | **12** |
| **Whole heart [mg]** | 150.7 ± 4.3 | 220.5 ± 12.5 * | 152.5 ± 6.2 | 224.2 ± 13.3 * |
| **Atria [mg]** | 8.6 ± 0.7 | 15.0 ± 1.6 | 8.4 ± 0.5 | 17.7 ± 3.9 * |
| **Ventricles [mg]** | 142.3 ± 4.6 | 205.5 ± 11.6 * | 144.1 ± 6.1 | 206.5 ± 10.6 * |
| ***Wet organ weights*** |  |  |  |  |
| **n** | **10-12** | **11-14** | **11-13** | **8-9** |
| **Lungs [mg]** | 147.6 ± 7.3 | 183.8 ± 20.3 | 150.5 ± 12.3 | 163.5 ± 20.3 |
| **Kidney [mg]** | 175.7 ± 3.5 | 171.7 ± 6.2 | 166.5 ± 4.0 | 164.7 ± 8.9 |
| **Spleen [mg]** | 81.8 ± 3.3 | 81.3 ± 3.7 | 85.0 ± 5.4 | 94.5 ± 4.9 |
| ***Dry organ weights*** |  |  |  |  |
| **n** | **10-11** | **11-13** | **11-12** | **8-9** |
| **Lungs [mg]** | 29.0 ± 0.9 | 35.4 ± 3.5 | 28.6 ± 0.9 | 42.1 ± 7.3 * |
| **Kidney [mg]** | 45.4 ± 1.3 | 43.6 ± 1.6 | 42.9 ± 0.9 | 40.8 ± 2.4 |
| ***Wet organ weights normalized to body weight [mg/g]*** | | |  |  |
| **n** | **11-12** | **14-15** | **12-13** | **10-12** |
| **Heart [mg]** | 4.8 ± 0.2 | 7.2 ± 0.4 * | 5.2 ± 0.2 | 7.5 ± 0.5 * |
| **Spleen [mg]** | 2.8 ± 0.1 | 2.8 ± 0.1 | 3.1 ± 0.2 | 3.3 ± 0.2 |
| ***Wet organ weights normalized to tibia length [mg/mm]*** | | |  |  |
| **n** | **10-11** | **10-11** | **10-11** | **8-9** |
| **Heart [mg]** | 7.8 ± 0.3 | 12.0 ± 0.6 * | 8.2 ± 0.4 | 12.2 ± 0.4 * |
| **Spleen [mg]** | 4.7 ± 0.1 | 4.5 ± 0.3 | 4.7 ± 0.4 | 5.4 ± 0.3 |

Mean ± SE, *p<0.05 vs. corresponding Sham, #p<0.05 vs. TAC - PAT; Two-way ANOVA with Sidak’s post hoc test

**Supplementary methods**

**Transverse aortic constriction (TAC) and PAT excision**

Mice were randomly assigned to either permanent banding of the aortic arch between the innominate artery and the right subclavian artery using a 7-0 thread and a 27 G place holder to induce LV afterload or to a sham operation, respectively. Surgeries were performed under deep anesthesia (medetomidin/midazolam/fentanyl; 0.5/5.0/0.05 mg/kg; intraperitoneally, i.p.). Anesthesia depth/surgical tolerance was confirmed by lack of blinking, whiskers movement and toe withdrawal effect. After subsequent intubation and fur removal as well as disinfection of the surgery field, the skin was opened parallel to the sternum via a 1 cm incision and the pectoral muscles bluntly separated. Ventilation throughout the procedure was assured via a tidal volume of 250 µl and 150 strokes/min using the Hugo Sachs Mini-Vent. The thorax was then opened in the first intercostal room and kept open via two 5-0 silk threads threaded around the first and the second rip, respectively. The thymus was bluntly severed, the perivascular fat and connective tissue surrounding the aortic arch between the innominate and the subclavian artery branch were carefully detached. Subsequently, a non-resorbable 7-0 thread was threaded around the arch between the innominate and the right subclavian artery branch and knotted around a 27 G place holder followed by the place holder removal. The intercostal space was closed by a single knot of the two 5-0 silk sutures which had served as retractors to keep the surgery field open beforehand. Sham animals underwent the same procedure without the transverse aortic constriction. Subsequently, a subset of sham and TAC mice was further subjected to PAT excision. Herein, the third intercostal room was opened by dissecting the intercostal muscle, which was kept open via two 5-0 silk threads threaded around the third and the fourth rip, respectively. The part of PAT, which is attached to the LV expanding from below the left atrium to the apex, was excised and the intercostal space was closed by single knot of the two 5-0 silk sutures. Skin was closed with single knot sutures using 5-0 silk. Anesthesia was antagonized by flumazenil/atipamezol (0.1/0.5 mg/kg; i.p.). For analgesia buprenorphine (0.05 mg/kg body weight; subcutaneously, s.c.) was injected immediately, 6 h, 24 h and 32 h after surgery. In addition, meloxicam (0.2 mg/kg; s.c.) was administered 24 h after surgery.

**Flow cytometry antibodies and gating strategies**

All obtained cell suspensions were rinsed, re-suspended in PBS with 1% BSA, subjected to CD16/CD32 Fc-block and incubated with antibodies against murine CD45 (30-F11, BioLegend), CD3 (17A2, BD Biosciences), CD4 (GK1.5, BD Biosciences), CD8a (53-6.7, Invitrogen), CD11b (M1/70, BioLegend), CD19 (eBio1D3, Invitrogen), CD64 (X54-5/7.1, BioLegend), CD115 (AFS98, Invitrogen), CD206 (C068C2, BioLegend), Ly6G (1A8, BioLegend), Ly6C (HK1.4, BioLegend), MHCII (M5/114.15.2, Invitrogen),Timd4 (RMT4-54, BioLegend).

Myeloid panels: Neutrophils were identified as CD45^+^ CD11b^+^ Ly6G^+^; classical monocytes as CD45^+^ CD11b^+^ CD115^+^ Ly6G^-^ CD64^-^ LY6C^high^; non-classical monocytes as CD45^+^ CD11b^+^ CD115^+^ Ly6G^-^ CD64^-^ LY6C^low^, or as CD45^+^ CD11b^+^ Ly6G^-^ CD64^-^ LY6C^high/low^ in the heart. LV resident and recruited macrophages (CD45^+^ CD11b^+^ Ly6G^-^ CD64^+^) were discriminated via their gating as Timd4^+^ MHCII^+/-^ or Timd4^-^ Ly6C^low/high^ MHCII^+/-^. Alternatively activated macrophages were identified by CD206 surface expression.

Lymphoid panels: Lymphoids were either roughly identified as CD45^+^ CD11b^-^ or subdivided further into CD3^+^CD4^+/-^CD8a^-/+^ T-cells and CD19^+^ B-cells. Data were acquired on FACS Canto II and Fortessa (BD Biosciences), and analyses were performed using the FloJo software (Ashland, USA).

**LV mRNA expression analyses**

TaqMan probes, purchased from Thermo Scientific: *Adrb1* Mm00431701_s1; *Agtr1a Mm00616371_m1; Ctgf* Mm01192933_g1; *Col Iα2* Mm00483888_m1; *Col IIIα1* Mm01254476_m1; *Gapdh* Mm99999915_g1; *Il-1b* Mm00434228_m1; *Il-6* Mm00446190_m1; *Mmp2* Mm00439498_m1; *Myh7* Mm00600555_m1*; Nppa* Mm01255747_g1; *Nppb* Mm01255770_g1; *Postn* Mm01284919_m1*; Tgfβ* Mm01178820_m1*; Tnfα* Mm00443258_m1; *Th* Mm00447557_m1.

**Immunohistochemistry**

For tyrosine hydroxylase (TH) staining, PAT excised during tissue sampling was fixed with 2% PFA, permeabilized with 0.5% triton X-100, and blocked with 5% BSA and rat anti-CD16/CD32 (553142, BD Biosciences). PAT was stained with rabbit anti-tyrosine hydroxylase (NB300-109, Novus; secondary antibody: Alexa Fluor 647 Donkey anti-rat IgG, 712-605-153, JacksonImmunoResearch) and goat anti-perilipin-1 (ab61682, Abcam; Alexa Fluor 488 Donkey anti-goat IgG, 705-545-003, JacksonImmunoResearch). Nuclei were stained with Hoechst (H3570, Invitrogen, Waltham, MA, USA). PAT was cleared and mounted with RapiClear 1.47 (RC147001, SUNJIN Lab). Tile scans of PAT were acquired in 10x magnification. The total length of sympathetic TH+ nerves within PAT was quantified using LAS X software.

For α-SMA and macrophage staining, acetone-fixed and -permeabilized LV transverse OCT sections were blocked with 1% BSA in PBS, and stained with FITC mouse anti-α-SMA (F3777, Sigma-Aldrich) or mouse anti-α-actinin (A7811, Sigma-Aldrich; secondary antibody: FITC donkey anti-mouse, 715-096-150, JacksonImmunoResearch, West Grove, PA, USA), rat anti-mouse CD68 (MCA 1957GA, Bio-Rad; Hercules, CA, USA; secondary antibody: Alexa Fluor 647 Donkey anti-rat IgG, 712-605-153, JacksonImmunoResearch) and goat anti-mouse CD206 (AF2535, R&D, Wiesbaden, Germany; secondary antibody: Rhodamine Red-X Donkey anti-goat, 705-295-147, JacksonImmunoResearch), respectively. Nuclei were stained with Hoechst (H3570, Invitrogen, Waltham, MA, USA). Myofibroblasts were manually counted (Image J) from tile scans of the whole section acquired in 10x magnification. Herein a α-SMA+ signal that surrounded a nucleus was identified as an individual cell i.e. a myofibroblast, and α-SMA + signals that clearly indicate the border of a lumen, suggestive of vessels and thereby vascular smooth muscle cells were excluded from the analyses. Macrophage populations were manually counted (Image J) in 8 fields of view (FoV) per section, acquired in 20x magnification, and averaged per section. CD68+CD206+ and CD68+CD206- subpopulations were quantified as percentage (%) of CD68+ cells.

**Cell culture**

To elaborate whether PAT exhibits pro-fibrotic signatures, cardiac fibroblasts (cFBs) were exposed to PAT protein extracted from unstressed mice and 12 wks TAC subjected mice and characterized based on cell viability, proliferation and collagen production [2, 3, 5]. cFBs were obtained from 12-weeks old C57BL/6J males (Charles River, Sulzfeld, Germany), as described previously [1, 4]. In brief, LV tissue pieces were fixed in 12-well culture plates with Dulbecco's modified eagle high glucose medium (DMEM; Gibco; Life Technologies, Darmstadt, Germany) containing 20% foetal bovine serum (FBS; Bio&Sell, Feucht, Germany) and 1% penicillin (P)/streptomycin (S) (Gibco; Life Technologies). Four weeks after seeding, outgrowing cFBs were harvested for further cell culture maintenance. PAT exposure experiments were performed at passage 7 with cFBs of n=3 individual donor mice with 3-6 wells per individual and condition. Herein, 5,000 cells/well were seeded in DMEM supplemented with 20% FBS and 1% P/S in 96-well plates. 7-8 h after seeding, media was removed, cells were washed once with 1x PBS, and then starved with DMEM containing 0.5% FBS and 1% P/S overnight (15 h). At the next day, cells were stimulated with starvation media containing PAT whole cell lysate at 10 ng/ml and 100 ng/ml, TGF-β (5 ng/ml) as positive control, and sole starving medium as negative control, for 72 h followed by measurement of cFB viability/metabolic activity, cell counts and collagen contents.

To assess cFB viability/metabolic activity, 20 μl of CellTiter 96^®^ AQ_ueous_ One Solution Reagent (Promega) was added to each well, followed by incubation at 37°C for 1 h and measurement of tetrazole to formazan reduction via absorbance at 490 nm. Cell count was assessed via absorbance measurement at 595 nm of 4% paraformaldehyde fixed, crystal violet- (Sigma-Aldrich Chemie GmbH) stained cells. To assess the collagen content, cells were fixed with cold methanol, stained with sirius red (Sigma-Aldrich Chemie GmbH) and absorbance was measured at 540 nm. Absorbances were read by the Spectra Max 340PC microplate reader (Molecular Device GmbH LLC, San Jose, CA, USA).

**References**

1. Matz I, Pappritz K, Springer J, Van Linthout S (2022) Left ventricle- and skeletal muscle-derived fibroblasts exhibit a differential inflammatory and metabolic responsiveness to interleukin-6. Front Immunol 13:947267 doi: 10.3389/fimmu.2022.947267

2. Pappritz K, Lin J, El-Shafeey M, Fechner H, Kuhl U, Alogna A, Spillmann F, Elsanhoury A, Schulz R, Tschope C, Van Linthout S (2022) Colchicine prevents disease progression in viral myocarditis via modulating the NLRP3 inflammasome in the cardiosplenic axis. ESC Heart Fail 9:925-941 doi: 10.1002/ehf2.13845

3. Pappritz K, Puhl SL, Matz I, Brauer E, Shia YX, El-Shafeey M, Koch SE, Miteva K, Mucha C, Duda GN, Petersen A, Steffens S, Tschope C, Van Linthout S (2023) Sex- and age-related differences in the inflammatory properties of cardiac fibroblasts: impact on the cardiosplenic axis and cardiac fibrosis. Front Cardiovasc Med 10:1117419 doi: 10.3389/fcvm.2023.1117419

4. Pappritz K, Savvatis K, Koschel A, Miteva K, Tschope C, Van Linthout S (2018) Cardiac (myo)fibroblasts modulate the migration of monocyte subsets. Sci Rep 8:5575 doi: 10.1038/s41598-018-23881-7

5. Spillmann F, Miteva K, Pieske B, Tschope C, Van Linthout S (2015) High-density lipoproteins reduce endothelial-to-mesenchymal transition. Arterioscler Thromb Vasc Biol 35:1774-1777 doi: 10.1161/ATVBAHA.115.305887

| **Suppl. table S3**: **Exact group sizes and calculated p-values for each experimental data set displayed in the main article** | | | | | | | |  |
| --- | --- | --- | --- | --- | --- | --- | --- | --- |
| **Fig. 1c Diameter** |  |  |  |  |  | |  |  |
| **8 wks** | Sham (n=6) vs. TAC (n=8) p=0.002 | Sham - PAT (n=6) vs. TAC - PAT (n=6) p=0.006 |  |  |  | |  |  |
| **12 wks** | Sham (n=6) vs. TAC (n=7) p=0.002 | Sham - PAT (n=5) vs. TAC - PAT (n=6) p=0.003 |  |  |  | |  |  |
|  |  |  |  |  |  | |  |  |
| **Fig. 1d left panel IVS;d** |  |  |  |  |  | |  |  |
| **1 wks** | Sham (n=10) vs. TAC (n=10) ns | Sham - PAT (n=9) vs. TAC - PAT (n=10) ns |  |  |  | |  |  |
| **4 wks** | Sham (n=11) vs. TAC (n=10) ns | Sham - PAT (n=12) vs. TAC - PAT (n=11) p=0.0041 |  |  |  | |  |  |
| **8 wks** | Sham (n=9) vs. TAC (n=13) p=0.0004 | Sham - PAT (n=11) vs. TAC - PAT (n=10) ns |  |  |  | |  |  |
| **12 wks** | Sham (n=10) vs. TAC (n=13) ns | Sham - PAT (n=11) vs. TAC - PAT (n=12) p=0.0012 | TAC vs. TAC - PAT ns |  |  | |  |  |
| **TAC** | 1 wk vs. 4 wks ns | 1 wk vs. 8 wks p=0.0127 | 1 wk vs. 12 wks p=0.0093 | 4 wk vs. 8 wks ns | 4 wk vs. 12 wks ns | | 8 wks vs. 12 wks ns |  |
| **TAC - PAT** | 1 wk vs. 4 wks ns | 1 wk vs. 8 wks ns | 1 wk vs. 12 wks p=0.0054 | 4 wk vs. 8 wks ns | 4 wk vs. 12 wks ns | | 8 wks vs. 12 wks ns |  |
|  |  |  |  |  |  | |  |  |
| **Fig. 1d middle panel LVAW;d** |  |  |  |  |  | |  |  |
| **1 wks** | Sham (n=10) vs. TAC (n=10) ns | Sham - PAT (n=9) vs. TAC - PAT (n=10) ns |  |  |  | |  |  |
| **4 wks** | Sham (n=11) vs. TAC (n=10) p=0.019 | Sham - PAT (n=12) vs. TAC - PAT (n=11) p=0.0028 |  |  |  | |  |  |
| **8 wks** | Sham (n=9) vs. TAC (n=13) p=0.055 | Sham - PAT (n=11) vs. TAC - PAT (n=10) p=0.0117 |  |  |  | |  |  |
| **12 wks** | Sham (n=10) vs. TAC (n=13) ns | Sham - PAT (n=11) vs. TAC - PAT (n=12) p=0.018 | TAC vs. TAC - PAT p<0.0001 |  |  | |  |  |
| **TAC** | 1 wk vs. 4 wks ns | 1 wk vs. 8 wks p=0.013 | 1 wk vs. 12 wks p=0.015 | 4 wk vs. 8 wks ns | 4 wk vs. 12 wks ns | | 8 wks vs. 12 wks ns |  |
| **TAC - PAT** | 1 wk vs. 4 wks ns | 1 wk vs. 8 wks p=0.0002 | 1 wk vs. 12 wks ns | 4 wk vs. 8 wks ns | 4 wk vs. 12 wks ns | | 8 wks vs. 12 wks ns |  |
|  |  |  |  |  |  | |  |  |
| **Fig. 1d right panel LVPW;d** |  |  |  |  |  | |  |  |
| **1 wks** | Sham (n=10) vs. TAC (n=10) ns | Sham - PAT (n=9) vs. TAC - PAT (n=10) ns |  |  |  | |  |  |
| **4 wks** | Sham (n=11) vs. TAC (n=10) ns | Sham - PAT (n=12) vs. TAC - PAT (n=11) p=0.0117 |  |  |  | |  |  |
| **8 wks** | Sham (n=9) vs. TAC (n=13) p=0.0033 | Sham - PAT (n=11) vs. TAC - PAT (n=10) p=0.0014 |  |  |  | |  |  |
| **12 wks** | Sham (n=10) vs. TAC (n=13) ns | Sham - PAT (n=11) vs. TAC - PAT (n=12) p=0.0114 | TAC vs. TAC - PAT ns |  |  | |  |  |
| **TAC** | 1 wk vs. 4 wks ns | 1 wk vs. 8 wks p=0.035 | 1 wk vs. 12 wks p=0.0090 | 4 wk vs. 8 wks ns | 4 wk vs. 12 wks ns | | 8 wks vs. 12 wks ns |  |
| **TAC - PAT** | 1 wk vs. 4 wks ns | 1 wk vs. 8 wks p=0.0019 | 1 wk vs. 12 wks ns | 4 wk vs. 8 wks ns | 4 wk vs. 12 wks ns | | 8 wks vs. 12 wks ns |  |
|  |  |  |  |  |  | |  |  |
|  |  |  |  |  |  | |  |  |
| **Fig. 1e E/A ratio** |  |  |  |  |  | |  |  |
| **8 wks** | Sham (n=9) vs. TAC (n=8) p=0.0104 | Sham - PAT (n=9) vs. TAC - PAT (n=7) p=0.0004 |  |  |  | |  |  |
| **12 wks** | Sham (n=8) vs. TAC (n=7) p=0.0015 | Sham - PAT (n=10) vs. TAC - PAT (n=8) p<0.0001 |  |  |  | |  |  |
|  |  |  |  |  |  | |  |  |
| **Fig. 1h left panel heart weight** |  |  |  |  |  | |  |  |
| **1 wks** | Sham (n=17) vs. TAC (n=18) ns | Sham - PAT (n=9) vs. TAC - PAT (n=11) ns |  |  |  | |  |  |
| **8 wks** | Sham (n=11) vs. TAC (n=15) p<0.0001 | Sham - PAT (n=12) vs. TAC - PAT (n=13) p<0.0001 |  |  |  | |  |  |
| **12 wks** | Sham (n=18) vs. TAC (n=18) p<0.0001 | Sham - PAT (n=14) vs. TAC - PAT (n=12) p<0.0001 | TAC vs. TAC - PAT p=0.003 |  |  | |  |  |
| **TAC** | 1 wk vs. 8 wks p<0.0001 | 1 wk vs. 12 wks p<0.0001 | 8 wks vs. 12 wks p=0.003 |  |  | |  |  |
| **TAC - PAT** | 1 wk vs. 8 wks p<0.0001 | 1 wk vs. 12 wks p<0.0001 | 8 wks vs. 12 wks ns |  |  | |  |  |
|  |  |  |  |  |  | |  |  |
| **Fig. 1h middle panel HW/BW** |  |  |  |  |  | |  |  |
| **1 wks** | Sham (n=17) vs. TAC (n=18) ns | Sham - PAT (n=9) vs. TAC - PAT (n=11) ns |  |  |  | |  |  |
| **8 wks** | Sham (n=11) vs. TAC (n=15) p<0.0001 | Sham - PAT (n=12) vs. TAC - PAT (n=13) p<0.0001 |  |  |  | |  |  |
| **12 wks** | Sham (n=18) vs. TAC (n=18) p<0.0001 | Sham - PAT (n=14) vs. TAC - PAT (n=12) p<0.0001 | TAC vs. TAC - PAT p=0.005 |  |  | |  |  |
| **TAC** | 1 wk vs. 8 wks p<0.0001 | 1 wk vs. 12 wks p<0.0001 | 8 wks vs. 12 wks p=0.02 |  |  | |  |  |
| **TAC - PAT** | 1 wk vs. 8 wks p=0.0004 | 1 wk vs. 12 wks p=0.025 | 8 wks vs. 12 wks ns |  |  | |  |  |
|  |  |  |  |  |  | |  |  |
| **Fig. 1h right panel HW/TL** |  |  |  |  |  | |  |  |
| **1 wks** | Sham (n=12) vs. TAC (n=15) ns | Sham - PAT (n=8) vs. TAC - PAT (n=9) ns |  |  |  | |  |  |
| **8 wks** | Sham (n=10) vs. TAC (n=11) p<0.0001 | Sham - PAT (n=11) vs. TAC - PAT (n=9) p<0.0001 |  |  |  | |  |  |
| **12 wks** | Sham (n=10) vs. TAC (n=10) p<0.0001 | Sham - PAT (n=10) vs. TAC - PAT (n=9) p<0.0001 |  |  |  | |  |  |
| **TAC** | 1 wk vs. 8 wks p<0.0001 | 1 wk vs. 12 wks p<0.0001 | 8 wks vs. 12 wks ns |  |  | |  |  |
| **TAC - PAT** | 1 wk vs. 8 wks p<0.0001 | 1 wk vs. 12 wks p<0.0001 | 8 wks vs. 12 wks ns |  |  | |  |  |
|  |  |  |  |  |  | |  |  |
| **Fig. 1i LV mass** |  |  |  |  |  | |  |  |
| **1 wks** | Sham (n=10) vs. TAC (n=10) ns | Sham - PAT (n=9) vs. TAC - PAT (n=10) ns |  |  |  | |  |  |
| **4 wks** | Sham (n=11) vs. TAC (n=10) ns | Sham - PAT (n=12) vs. TAC - PAT (n=11) p=0.013 |  |  |  | |  |  |
| **8 wks** | Sham (n=9) vs. TAC (n=13) p<0.0001 | Sham - PAT (n=11) vs. TAC - PAT (n=10) p<0.0001 |  |  |  | |  |  |
| **12 wks** | Sham (n=10) vs. TAC (n=13) p<0.0001 | Sham - PAT (n=11) vs. TAC - PAT (n=12) p<0.0001 | TAC vs. TAC - PAT p=0.0019 |  |  | |  |  |
| **TAC** | 1 wk vs. 4 wks p=0.015 | 1 wk vs. 8 wks p<0.0001 | 1 wk vs. 12 wks p<0.0001 | 4 wk vs. 8 wks p=0.0052 | 4 wk vs. 12 wks p<0.0001 | | 8 wks vs. 12 wks ns |  |
| **TAC - PAT** | 1 wk vs. 4 wks p=0.0362 | 2 wk vs. 8 wks p<0.0001 | 2 wk vs. 12 wks p<0.0001 | 4 wk vs. 8 wks p=0.0057 | 4 wk vs. 12 wks ns | | 8 wks vs. 12 wks ns |  |
|  |  |  |  |  |  | |  |  |
| **Fig. 2a 1st panel LVID;d** |  |  |  |  |  | |  |  |
| **1 wks** | Sham (n=10) vs. TAC (n=10) ns | Sham - PAT (n=9) vs. TAC - PAT (n=10) ns |  |  |  | |  |  |
| **4 wks** | Sham (n=11) vs. TAC (n=10) ns | Sham - PAT (n=12) vs. TAC - PAT (n=11) ns |  |  |  | |  |  |
| **8 wks** | Sham (n=9) vs. TAC (n=13) p=0.0007 | Sham - PAT (n=11) vs. TAC - PAT (n=10) p<0.0001 |  |  |  | |  |  |
| **12 wks** | Sham (n=10) vs. TAC (n=13) p<0.0001 | Sham - PAT (n=11) vs. TAC - PAT (n=12) ns | TAC vs. TAC - PAT p<0.0001 |  |  | |  |  |
| **TAC** | 1 wk vs. 4 wks ns | 1 wk vs. 8 wks p<0.0001 | 1 wk vs. 12 wks p<0.0001 | 4 wk vs. 8 wks p=0.041 | 4 wk vs. 12 wks p<0.0001 | | 8 wks vs. 12 wks ns |  |
| **TAC - PAT** | 1 wk vs. 4 wks ns | 1 wk vs. 8 wks p=0.0002 | 1 wk vs. 12 wks p=0.0105 | 4 wk vs. 8 wks p=0.023 | 4 wk vs. 12 wks ns | | 8 wks vs. 12 wks ns |  |
|  |  |  |  |  |  | |  |  |
| **Fig. 2a second panel LVID;s** |  |  |  |  |  | |  |  |
| **1 wks** | Sham (n=10) vs. TAC (n=10) ns | Sham - PAT (n=9) vs. TAC - PAT (n=10) ns |  |  |  | |  |  |
| **4 wks** | Sham (n=11) vs. TAC (n=10) ns | Sham - PAT (n=12) vs. TAC - PAT (n=11) ns |  |  |  | |  |  |
| **8 wks** | Sham (n=9) vs. TAC (n=13) p<0.0001 | Sham - PAT (n=11) vs. TAC - PAT (n=10) p<0.0001 | TAC vs. TAC - PAT ns |  |  | |  |  |
| **12 wks** | Sham (n=10) vs. TAC (n=13) p<0.0001 | Sham - PAT (n=11) vs. TAC - PAT (n=12) p=0.0072 | TAC vs. TAC - PAT p<0.0001 |  |  | |  |  |
| **TAC** | 1 wk vs. 4 wks p=0.043 | 1 wk vs. 8 wks p<0.0001 | 1 wk vs. 12 wks p<0.0001 | 4 wk vs. 8 wks ns | 4 wk vs. 12 wks p<0.0001 | | 8 wks vs. 12 wks p=0.04 |  |
| **TAC - PAT** | 1 wk vs. 4 wks ns | 1 wk vs. 8 wks p=0.0002 | 1 wk vs. 12 wks p=0.024 | 4 wk vs. 8 wks ns | 4 wk vs. 12 wks ns | | 8 wks vs. 12 wks ns |  |
|  |  |  |  |  |  | |  |  |
| **Fig. 2a third panel LVvol;d** |  |  |  |  |  | |  | |
| **1 wks** | Sham (n=10) vs. TAC (n=10) ns | Sham - PAT (n=9) vs. TAC - PAT (n=10) ns |  |  |  | |  | |
| **4 wks** | Sham (n=11) vs. TAC (n=10) ns | Sham - PAT (n=12) vs. TAC - PAT (n=11) ns |  |  |  | |  | |
| **8 wks** | Sham (n=9) vs. TAC (n=13) p=0.0003 | Sham - PAT (n=11) vs. TAC - PAT (n=10) p=0.0002 |  |  |  | |  | |
| **12 wks** | Sham (n=10) vs. TAC (n=13) p<0.0001 | Sham - PAT (n=11) vs. TAC - PAT (n=12) ns | TAC vs. TAC - PAT p<0.0001 |  |  | |  | |
| **TAC** | 1 wk vs. 4 wks ns | 1 wk vs. 8 wks p<0.0001 | 1 wk vs. 12 wks p<0.0001 | 4 wk vs. 8 wks p=0.024 | 4 wk vs. 12 wks p<0.0001 | | 8 wks vs. 12 wks p=0.0195 | |
| **TAC - PAT** | 1 wk vs. 4 wks ns | 1 wk vs. 8 wks p=0.0004 | 1 wk vs. 12 wks p=0.025 | 4 wk vs. 8 wks p=0.024 | 4 wk vs. 12 wks ns | | 8 wks vs. 12 wks ns | |
|  |  |  |  |  |  | |  | |
| **Fig. 2a fourth panel LVvol;s** |  |  |  |  |  | |  | |
| **1 wks** | Sham (n=10) vs. TAC (n=10) ns | Sham - PAT (n=9) vs. TAC - PAT (n=10) ns |  |  |  | |  | |
| **4 wks** | Sham (n=11) vs. TAC (n=10) ns | Sham - PAT (n=12) vs. TAC - PAT (n=11) ns |  |  |  | |  | |
| **8 wks** | Sham (n=9) vs. TAC (n=13) p<0.0001 | Sham - PAT (n=11) vs. TAC - PAT (n=10) p<0.0001 |  |  |  | |  | |
| **12 wks** | Sham (n=10) vs. TAC (n=13) p<0.0001 | Sham - PAT (n=11) vs. TAC - PAT (n=12) p=0.022 | TAC vs. TAC - PAT p<0.0001 |  |  | |  | |
| **TAC** | 1 wk vs. 4 wks ns | 1 wk vs. 8 wks p<0.0001 | 1 wk vs. 12 wks p<0.0001 | 4 wk vs. 8 wks ns | 4 wk vs. 12 wks p<0.0001 | | 8 wks vs. 12 wks p=0.0021 | |
| **TAC - PAT** | 1 wk vs. 4 wks ns | 1 wk vs. 8 wks p=0.0011 | 1 wk vs. 12 wks ns | 4 wk vs. 8 wks ns | 4 wk vs. 12 wks ns | | 8 wks vs. 12 wks ns | |
|  |  |  |  |  |  | |  | |
| **Fig. 2d upper panel EF** |  |  |  |  |  | |  | |
| **1 wks** | Sham (n=10) vs. TAC (n=10) ns | Sham - PAT (n=9) vs. TAC - PAT (n=10) ns |  |  |  | |  | |
| **4 wks** | Sham (n=11) vs. TAC (n=10) ns | Sham - PAT (n=12) vs. TAC - PAT (n=11) p=0.0016 |  |  |  | |  | |
| **8 wks** | Sham (n=9) vs. TAC (n=13) p<0.0001 | Sham - PAT (n=11) vs. TAC - PAT (n=10) p<0.0001 |  |  |  | |  | |
| **12 wks** | Sham (n=10) vs. TAC (n=13) p<0.0001 | Sham - PAT (n=11) vs. TAC - PAT (n=12) p=0.0009 | TAC vs. TAC - PAT p=0.015 |  |  | |  | |
| **TAC** | 1 wk vs. 4 wks ns | 1 wk vs. 8 wks p=0.004 | 1 wk vs. 12 wks p<0.0001 | 4 wk vs. 8 wks ns | 4 wk vs. 12 wks p=0.0116 | | 8 wks vs. 12 wks ns | |
| **TAC - PAT** | 1 wk vs. 4 wks ns | 1 wk vs. 8 wks p=0.0056 | 1 wk vs. 12 wks ns | 4 wk vs. 8 wks ns | 4 wk vs. 12 wks ns | | 8 wks vs. 12 wks ns | |
|  |  |  |  |  |  | |  | |
| **Fig. 2d lower panel FS** |  |  |  |  |  | |  | |
| **1 wks** | Sham (n=10) vs. TAC (n=10) ns | Sham - PAT (n=9) vs. TAC - PAT (n=10) p=0.042 |  |  |  | |  | |
| **4 wks** | Sham (n=11) vs. TAC (n=10) ns | Sham - PAT (n=12) vs. TAC - PAT (n=11) p=0.0009 |  |  |  | |  | |
| **8 wks** | Sham (n=9) vs. TAC (n=13) p<0.0001 | Sham - PAT (n=11) vs. TAC - PAT (n=10) p<0.0001 |  |  |  | |  | |
| **12 wks** | Sham (n=10) vs. TAC (n=13) p<0.0001 | Sham - PAT (n=11) vs. TAC - PAT (n=12) p=0.0007 | TAC vs. TAC - PAT p=0.046 |  |  | |  | |
| **TAC** | 1 wk vs. 4 wks ns | 1 wk vs. 8 wks p=0.012 | 1 wk vs. 12 wks p<0.0001 | 4 wk vs. 8 wks ns | 4 wk vs. 12 wks p=0.032 | | 8 wks vs. 12 wks ns | |
| **TAC - PAT** | 1 wk vs. 4 wks ns | 1 wk vs. 8 wks ns | 1 wk vs. 12 wks ns | 4 wk vs. 8 wks ns | 4 wk vs. 12 wks ns | | 8 wks vs. 12 wks ns | |
|  |  |  |  |  |  | |  | |
| **Fig. 2e left panel *Myh7*** |  |  |  |  |  | |  | |
| **1 wks** | Sham (n=8) vs. TAC (n=9) ns | Sham - PAT (n=5) vs. TAC - PAT (n=6) ns |  |  |  | |  | |
| **8 wks** | Sham (n=7) vs. TAC (n=7) ns | Sham - PAT (n=7) vs. TAC - PAT (n=5) ns |  |  |  | |  | |
| **12 wks** | Sham (n=9) vs. TAC (n=10) p<0.0001 | Sham - PAT (n=9) vs. TAC - PAT (n=8) ns | TAC vs. TAC - PAT p=0.0002 |  |  | |  | |
| **TAC** | 1 wk vs. 8 wks ns | 1 wk vs. 12 wks p<0.0001 | 8 wks vs. 12 wks p<0.0001 |  |  | |  | |
| **TAC - PAT** | 1 wk vs. 8 wks ns | 1 wk vs. 12 wks ns | 8 wks vs. 12 wks ns |  |  | |  | |
|  |  |  |  |  |  | |  | |
| **Fig. 2e middle panel *Nppa*** |  |  |  |  |  | |  | |
| **1 wks** | Sham (n=8) vs. TAC (n=9) p=0.019 | Sham - PAT (n=5) vs. TAC - PAT (n=6) ns |  |  |  | |  | |
| **8 wks** | Sham (n=7) vs. TAC (n=7) ns | Sham - PAT (n=7) vs. TAC - PAT (n=5) ns | TAC vs. TAC - PAT ns |  |  | |  | |
| **12 wks** | Sham (n=9) vs. TAC (n=10) p=0.003 | Sham - PAT (n=9) vs. TAC - PAT (n=8) ns | TAC vs. TAC - PAT p=0.069 |  |  | |  | |
| **TAC** | 1 wk vs. 8 wks p=0.026 | 1 wk vs. 12 wks ns | 8 wks vs. 12 wks p=0.0196 |  |  | |  | |
| **TAC - PAT** | 1 wk vs. 8 wks ns | 1 wk vs. 12 wks ns | 8 wks vs. 12 wks ns |  |  | |  | |
|  |  |  |  |  |  | |  | |
| **Fig. 2e right panel *Nppb*** |  |  |  |  |  | |  | |
| **1 wks** | Sham (n=8) vs. TAC (n=9) ns | Sham - PAT (n=5) vs. TAC - PAT (n=6) ns |  |  |  | |  | |
| **8 wks** | Sham (n=7) vs. TAC (n=7) p<0.0001 | Sham - PAT (n=7) vs. TAC - PAT (n=5) ns | TAC vs. TAC - PAT p=0.0005 |  |  | |  | |
| **12 wks** | Sham (n=9) vs. TAC (n=10) ns | Sham - PAT (n=9) vs. TAC - PAT (n=8) ns | TAC vs. TAC - PAT p=0.028 |  |  | |  | |
| **TAC** | 1 wk vs. 8 wks p=0.0006 | 1 wk vs. 12 wks ns | 8 wks vs. 12 wks p=0.0008 |  |  | |  | |
| **TAC - PAT** | 1 wk vs. 8 wks ns | 1 wk vs. 12 wks p=0.006 | 8 wks vs. 12 wks ns |  |  | |  | |
|  |  |  |  |  |  | |  | |
| **Fig. 3a CD64+** |  |  |  |  |  | |  | |
| **1 wks** | Sham (n=7) vs. TAC (n=7) ns | Sham - PAT (n=6) vs. TAC - PAT (n=5) ns | TAC vs. TAC - PAT ns |  |  | |  | |
| **8 wks** | Sham (n=7) vs. TAC (n=7) ns | Sham - PAT (n=6) vs. TAC - PAT (n=7) ns | TAC vs. TAC - PAT ns |  |  | |  | |
| **12 wks** | Sham (n=8) vs. TAC (n=9) ns | Sham - PAT (n=8) vs. TAC - PAT (n=7) ns | TAC vs. TAC - PAT ns |  |  | |  | |
| **TAC** | 1 wk vs. 8 wks ns | 1 wk vs. 12 wks ns | 8 wks vs. 12 wks ns |  |  | |  | |
| **TAC - PAT** | 1 wk vs. 8 wks ns | 1 wk vs. 12 wks p=0.0006 | 8 wks vs. 12 wks ns |  |  | |  | |
|  |  |  |  |  |  | |  | |
| **Fig. 3b TIMD4-** |  |  |  |  |  | |  | |
| **1 wks** | Sham (n=7) vs. TAC (n=7) p=0.0097 | Sham - PAT (n=6) vs. TAC - PAT (n=5) p=0.0089 | TAC vs. TAC - PAT ns |  |  | |  | |
| **8 wks** | Sham (n=7) vs. TAC (n=7) ns | Sham - PAT (n=6) vs. TAC - PAT (n=7) ns | TAC vs. TAC - PAT ns |  |  | |  | |
| **12 wks** | Sham (n=8) vs. TAC (n=9) ns | Sham - PAT (n=8) vs. TAC - PAT (n=7) ns | TAC vs. TAC - PAT ns |  |  | |  | |
| **TAC** | 1 wk vs. 8 wks p=0.0062 | 1 wk vs. 12 wks p=0.0214 | 8 wks vs. 12 wks ns |  |  | |  | |
| **TAC - PAT** | 1 wk vs. 8 wks p=0.0193 | 1 wk vs. 12 wks p<0.0001 | 8 wks vs. 12 wks ns |  |  | |  | |
|  |  |  |  |  |  | |  | |
| **Fig. 3c TIMD4- Ly6ClowMHCII-** |  |  |  |  |  | |  | |
| **1 wks** | Sham (n=7) vs. TAC (n=7) p=0.0008 | Sham - PAT (n=6) vs. TAC - PAT (n=5) p<0.0001 | TAC vs. TAC - PAT p=0.0084 |  |  | |  | |
| **8 wks** | Sham (n=7) vs. TAC (n=7) ns | Sham - PAT (n=6) vs. TAC - PAT (n=7) ns | TAC vs. TAC - PAT ns |  |  | |  | |
| **12 wks** | Sham (n=8) vs. TAC (n=9) ns | Sham - PAT (n=8) vs. TAC - PAT (n=7) ns | TAC vs. TAC - PAT ns |  |  | |  | |
| **TAC** | 1 wk vs. 8 wks p=0.0099 | 1 wk vs. 12 wks p=0.015 | 8 wks vs. 12 wks ns |  |  | |  | |
| **TAC - PAT** | 1 wk vs. 8 wks p<0.0001 | 1 wk vs. 12 wks p<0.0001 | 8 wks vs. 12 wks ns |  |  | |  | |
|  |  |  |  |  |  | |  | |
| **Fig. 3d TIMD4- Ly6ChighMHCII+** |  |  |  |  |  | |  | |
| **1 wks** | Sham (n=7) vs. TAC (n=7) p=0.0095 | Sham - PAT (n=6) vs. TAC - PAT (n=5) ns | TAC vs. TAC - PAT p=0.027 |  |  | |  | |
| **8 wks** | Sham (n=7) vs. TAC (n=7) ns | Sham - PAT (n=6) vs. TAC - PAT (n=7) ns | TAC vs. TAC - PAT ns |  |  | |  | |
| **12 wks** | Sham (n=8) vs. TAC (n=9) ns | Sham - PAT (n=8) vs. TAC - PAT (n=7) ns | TAC vs. TAC - PAT ns |  |  | |  | |
| **TAC** | 1 wk vs. 8 wks p<0.0001 | 1 wk vs. 12 wks p=0.0015 | 8 wks vs. 12 wks ns |  |  | |  | |
| **TAC - PAT** | 1 wk vs. 8 wks ns | 1 wk vs. 12 wks ns | 8 wks vs. 12 wks ns |  |  | |  | |
|  |  |  |  |  |  | |  | |
| **Fig. 3e Ly6Chigh** |  |  |  |  |  | |  | |
| **1 wks** | Sham (n=7) vs. TAC (n=7) p=0.0095 | Sham - PAT (n=6) vs. TAC - PAT (n=5) ns | TAC vs. TAC - PAT p=0.027 |  |  | |  | |
| **8 wks** | Sham (n=7) vs. TAC (n=7) ns | Sham - PAT (n=6) vs. TAC - PAT (n=7) ns | TAC vs. TAC - PAT ns |  |  | |  | |
| **12 wks** | Sham (n=8) vs. TAC (n=9) ns | Sham - PAT (n=8) vs. TAC - PAT (n=7) ns | TAC vs. TAC - PAT ns |  |  | |  | |
| **TAC** | 1 wk vs. 8 wks p<0.0001 | 1 wk vs. 12 wks p=0.0015 | 8 wks vs. 12 wks ns |  |  | |  | |
| **TAC - PAT** | 1 wk vs. 8 wks ns | 1 wk vs. 12 wks ns | 8 wks vs. 12 wks ns |  |  | |  | |
|  |  |  |  |  |  | |  | |
| **Fig. 5a left panel *Il-6*** |  |  |  |  |  | |  | |
| **8 wks** | Sham (n=7) vs. TAC (n=7) ns | Sham - PAT (n=7) vs. TAC - PAT (n=5) ns | TAC vs. TAC - PAT ns |  |  | |  | |
| **12 wks** | Sham (n=9) vs. TAC (n=9) p<0.0001 | Sham - PAT (n=8) vs. TAC - PAT (n=9) ns | TAC vs. TAC - PAT p=0.001 |  |  | |  | |
| **TAC** | 8 wks vs. 12 wks p=0.011 |  |  |  |  | |  | |
| **TAC - PAT** | 8 wks vs. 12 wks ns |  |  |  |  | |  | |
|  |  |  |  |  |  | |  | |
| **Fig. 5a right panel *Ctgf*** |  |  |  |  |  | |  | |
| **8 wks** | Sham (n=7) vs. TAC (n=7) ns | Sham - PAT (n=7) vs. TAC - PAT (n=5) ns | TAC vs. TAC - PAT ns |  |  | |  | |
| **12 wks** | Sham (n=9) vs. TAC (n=9) p=0.0002 | Sham - PAT (n=8) vs. TAC - PAT (n=9) ns | TAC vs. TAC - PAT p=0.0008 |  |  | |  | |
| **TAC** | 8 wks vs. 12 wks p=0.005 |  |  |  |  | |  | |
| **TAC - PAT** | 8 wks vs. 12 wks ns |  |  |  |  | |  | |
|  |  |  |  |  |  | |  | |
| **Fig. 5c LV interstitial fibrosis** |  |  |  |  |  | |  | |
| **8 wks** | Sham (n=5) vs. TAC (n=7) p=0.0015 | Sham - PAT (n=6) vs. TAC - PAT (n=5) ns | TAC vs. TAC - PAT ns |  |  | |  | |
| **12 wks** | Sham (n=7) vs. TAC (n=7) p=0.008 | Sham - PAT (n=5) vs. TAC - PAT (n=6) ns | TAC vs. TAC - PAT ns |  |  | |  | |
| **TAC** | 8 wks vs. 12 wks ns |  |  |  |  | |  | |
| **TAC - PAT** | 8 wks vs. 12 wks ns |  |  |  |  | |  | |
|  |  |  |  |  |  | |  | |
| **Fig. 5d perivascular fibrosis** |  |  |  |  |  | |  | |
| **8 wks** | TAC (n=6) vs. TAC - PAT (n=5) ns |  |  |  |  | |  | |
| **12 wks** | TAC (n=7) vs. TAC - PAT (n=5) p=0.018 |  |  |  |  | |  | |
| **TAC** | 8 wks vs. 12 wks ns |  |  |  |  | |  | |
| **TAC - PAT** | 8 wks vs. 12 wks ns |  |  |  |  | |  | |
|  |  |  |  |  |  | |  | |
| **Fig. 5e Microscar collagen** |  |  |  |  |  | |  | |
| **8 wks** | TAC (n=5) vs. TAC - PAT (n=4) ns |  |  |  |  | |  | |
| **12 wks** | TAC (n=5) vs. TAC - PAT (n=6) p=0.037 |  |  |  |  | |  | |
| **TAC** | 8 wks vs. 12 wks ns |  |  |  |  | |  | |
| **TAC - PAT** | 8 wks vs. 12 wks ns |  |  |  |  | |  | |
|  |  |  |  |  |  | |  | |
| **Fig. 6a Myofibroblasts** |  |  |  |  |  | |  | |
| **12 wks** | Sham (n=7) vs. TAC (n=8) p=0.019 | Sham - PAT (n=7) vs. TAC - PAT (n=4) p=0.059 | TAC vs. TAC - PAT ns |  |  | |  | |
|  |  |  |  |  |  | |  | |
| **Fig. 6b Postn** |  |  |  |  |  |  |  |  |
| **12 wks** | Sham (n=6) vs. TAC (n=8) p=0.0015 | Sham - PAT (n=6) vs. TAC - PAT (n=4) ns | TAC vs. TAC - PAT p=0.013 |  |  |  |  |  |
|  |  |  |  |  |  |  |  |  |
| **Fig. 6d left panel CD68+** |  |  |  |  |  | |  | |
| **12 wks** | Sham (n=5) vs. TAC (n=5) p=0.001 | Sham - PAT (n=5) vs. TAC - PAT (n=3) p=0.03 | TAC vs. TAC - PAT ns |  |  | |  | |
|  |  |  |  |  |  | |  | |
| **Fig. 6d middle panel CD68+CD206+** |  |  |  |  |  |  |  |  |
| **12 wks** | Sham (n=5) vs. TAC (n=5) p=0.0014 | Sham - PAT (n=5) vs. TAC - PAT (n=3) ns | TAC vs. TAC - PAT ns |  | |  |  |  |
|  |  |  |  |  | |  |  |  |
| **Fig. 6e CD68+CD206+** |  |  |  |  | |  |  |  |
| **12 wks** | TAC (n=5) vs. TAC - PAT (n=4) p=0.0011 |  |  |  | |  |  |  |
|  |  |  |  |  | |  |  |  |
| **Fig. 7b Cell viability** |  |  |  |  | |  |  |  |
| **72 h** | Ctrl vs. TGF-b p=0.041 | Ctrl vs. 10 ng PAT protein p=0.049 | Ctr vs. 100 ng PAT protein p=0.001 |  |  |  |  |  |
|  |  | TGF-b vs. 10 ng PAT protein ns | TGFb vs. 100 ng PAT protein ns |  |  |  |  |  |
| **Fig. 7c Collagen content** |  |  |  |  |  |  |  |  |
| **72 h** | Ctrl vs. TGF-b n=0.02 | Ctrl vs. 10 ng PAT protein ns | Ctr vs. 100 ng PAT protein p=<0.0001 |  |  |  |  |  |
|  |  | TGF-b vs. 10 ng PAT protein ns | TGFb vs. 100 ng PAT protein p=0.018 |  |  |  |  |  |
|  |  |  |  |  |  |  |  |  |
| **Fig. 7d Cell counts** |  |  |  |  | |  |  |  |
| **72 h** | Ctrl vs. TGF-b p=0.049 | Ctrl vs. 10 ng PAT protein p=0.094 | Ctr vs. 100 ng PAT protein p<0.0001 |  |  |  |  |  |
|  |  | TGF-b vs. 10 ng PAT protein ns | TGFb vs. 100 ng PAT protein p<0.0001 |  |  |  |  |  |
|  |  |  |  |  | |  |  |  |
| **Fig. 7h TGF-b1** |  |  |  |  | |  |  |  |
| **1 wk** | Sham (n=8) vs. TAC (n=14) ns | Sham - PAT (n=8) vs. TAC - PAT (n=11) ns | TAC vs. TAC - PAT ns |  | |  |  |  |
